# Biodegradability of Chromium-Free Finished Leather Through ISO 14855-1:2012 and ISO 20136:2020: A Multi-Faceted Analytical Approach

**DOI:** 10.3390/polym18192389

**Published:** 2026-09-30

**Authors:** Alberto Vico, María I. Maestre-López, Jose Miguel Carot-Sierra, Irene Lifante-Martinez, Elena Orgilés-Calpena, Guillermo Grindlay, Marcelo Bertazzo

**Affiliations:** 1 Footwear Technological Institute (INESCOP), C/Alemania 102—Polígono Campo Alto, 03600 Elda, Spain; avico@inescop.es (A.V.); imaestre@inescop.es (M.I.M.-L.); ilifante@inescop.es (I.L.-M.); eorgiles@inescop.es (E.O.-C.); 2 Department of Applied Statistics, Operational Research, and Quality, Universitat Politècnica de València, Camino de Vera s/n, 46022 Valencia, Spain; jcarot@eio.upv.es; 3 Department of Analytical Chemistry, Nutrition, and Food Sciences, University of Alicante, Crta\ San Vicente del Raspeig, s/n, 03080 San Vicente del Raspeig, Alicante, Spain

**Keywords:** chromium-free leather, collagen-based polymeric materials, natural polymers, biodegradation, compostability, ecotoxicity, infrared spectroscopy

## Abstract

While various methodologies have been used to evaluate leather biodegradability, limited data exist regarding their respective strengths, limitations and complementarity, especially for finished leathers produced using chrome-free tanning systems. Consequently, this work assesses the biodegradation of eight commercial eco-friendly tanned finished leathers (phenolic-, zeolite-, aluminium- and titanium-based treatments) using two standardised laboratory approaches, ISO 14855-1:2012 (controlled composting) and ISO 20136:2020 (liquid-phase biodegradation), together with physicochemical characterisation and phytotoxicity/ecotoxicity endpoints. The results showed that biodegradation generally occurred faster under ISO 20136:2020 conditions, mainly because this assay uses ground leather shavings and an aqueous, well-mixed medium, whereas ISO 14855-1:2012 evaluates mineralisation of larger leather pieces in a compost matrix. Phenolic- and titanium-tanned leathers showed consistently high biodegradation in both assays, whereas the aluminium-tanned leather biodegraded efficiently under ISO 14855-1:2012 but showed limited biodegradation under ISO 20136:2020, highlighting that assay conditions can reveal constraints related to the complete material architecture, collagen stabilisation, effective accessibility and method-specific exposure conditions rather than to a single leaching mechanism. Phytotoxicity and ecotoxicity assessments indicated low environmental impact of the degradation products under the tested conditions. Overall, the combined use of both standards provides a more comprehensive and industrially useful framework for assessing the end-of-life behaviour of finished leathers than either method alone.

## 1. Introduction

The introduction of new, environmentally friendly leather products into the market is experiencing significant growth, particularly among major companies supplying raw materials to tanneries. As a key player in the sustainable use of meat industry byproducts, leather production utilises collagen-based biomaterials, processing over 20 million cattle hides annually [1]. The global leather market was valued at approximately 375.57 billion USD in 2022 and is projected to reach 959.23 billion USD by 2032, reflecting an annual growth rate of 8.8% driven by innovations in sustainability [2]. At the same time, the leather and footwear industries are undergoing a profound transformation to meet the requirements of the green transition promoted by the European Union (EU). In line with these goals, the EU has established, among other measures, a ban on the disposal of textile waste in landfills and an obligation for separate waste collection and sorting by December 2024 [3]. In this regulatory and industrial context, reliable end-of-life assessment tools are increasingly needed to support material selection, product development and waste-management strategies.

In compliance with EU environmental regulations, the development of new tanning technologies is significantly improving the sustainability of leather production. These approaches reduce environmental impact and enhance material properties using chrome-free alternatives, which lessen the hazardous effects associated with traditional tanning methods. Examples of these innovative techniques include oxidised sodium lignosulfonate and polycarboxylate–aluminium–zirconium complexes [4]. These new technologies can reduce total organic carbon and biotoxicity in wastewater by as much as 43.4% compared with conventional methods, lowering the organic load and improving wastewater quality [4,5]. These agents can also deliver competitive performance metrics and physical properties comparable to chrome-tanned leather, helping maintain durability and quality. Furthermore, the reliance on biomass-derived agents encourages the use of renewable resources, aligns with a circular economy model and promotes a more sustainable leather production framework [6]. However, industrial adoption requires not only process-level improvements but also evidence on how finished commercial leathers behave at the end of life, where tanning, retanning, fatliquoring and finishing chemistries may jointly influence biodegradation.

A key aspect of leather’s sustainability is its biodegradability. Nevertheless, the properties of finished leathers have a major influence on their biodegradation depending on the chemicals used in their manufacturing process [7,8]. Eco-friendly options have demonstrated improved biodegradability performance relative to traditional Cr-based tanning methods [9,10], but the challenge remains to balance functional performance, durability and biodegradation in leather production to improve waste recycling and end-of-life management. Several approaches have been proposed in the literature for assessing leather biodegradability at a laboratory scale under both composting and liquid-phase conditions [8,11]. For composting studies, researchers frequently apply ISO 14855-1:2012, originally developed for plastics, because no equivalent leather-specific compostability standard is currently available [12]. Although this approach is time-consuming and requires careful control of compost quality and maturity, it provides a standardised framework for evaluating ultimate aerobic mineralization under controlled thermophilic composting conditions rather than a direct simulation of uncontrolled natural disposal. For example, Sardroudi [11] investigated the composting capacity of various leathers and leatherette materials according to the disintegration under composting conditions specified in ISO 20200:2023 [13]. They found that bovine leather samples fully degraded within 35 days, while leatherette samples showed no degradation even after 90 days due to the presence of non-biodegradable materials such as polyurethane and PVC. Similarly, Vico [10] studied the effect of different finishing products individually impregnated into leathers on biodegradation, using the ISO 14855-1:2012 standard [12]. In that study, collagen was used as a positive control and produced results similar to cellulose, the standard positive control in the ISO14855-1 test. Their research indicated that leather with bio-based polyurethane (79.37%), acrylic (84.20%) and wax (85.09%) finishes were biodegradable, whereas traditional chemical finishes such as isocyanate (57.30%) and nitrocellulose lacquer (52.91%) showed only moderate biodegradation levels. Alternatively, leather biodegradation has been investigated in the liquid phase due to its methodological simplicity and the significant reduction in testing time required compared with composting approaches (33 vs. 90 days). This method is particularly suitable for assessing the degradation potential of leathers derived from different tanning processes within relatively short timeframes, in accordance with ISO 20136:2020 [14], the first international standard specifically developed to evaluate leather biodegradability. Unlike composting methods, which require testing conditions at 58 °C, ISO 20136:2020 is conducted at approximately ambient temperature (23 °C) in an aqueous medium. Therefore, it should be interpreted as a standardised comparative biodegradation assay for leather, not as a direct representation of all possible environmental disposal scenarios.

While both composting and liquid-phase studies are valuable tools for assessing leather biodegradation, limited data are still available on the extent to which these approaches provide consistent, divergent or complementary information when applied to a representative set of commercially finished leathers produced using eco-friendly tanning systems. Therefore, the aim of this work is to assess the biodegradation of finished leather products using ISO 14855-1:2012, as a controlled composting assay originally developed for plastic materials, and ISO 20136:2020, as a leather-specific liquid-phase biodegradation assay. The objective is not to establish one methodology as universally superior, but to identify where both approaches converge or diverge and how their combined use may support industrial screening, formulation decisions and end-of-life assessment. For this purpose, leather samples were characterised using a multi-faceted analytical approach including attenuated total reflection infrared spectroscopy (ATR-FTIR), laser direct infrared spectroscopy (LDIR) and mercury porosimetry. Additionally, both ecotoxicity and phytotoxicity studies were conducted to evaluate the potential environmental impact of leather biodegradation byproducts using both standards.

## 2. Materials and Methods

### 2.1. Leather Samples

This study aimed to evaluate the biodegradation properties of leathers treated with different eco-friendly tanning and retanning agents, as summarised in Table 1. The leathers were subjected to a variety of Cr-free processing techniques, including zeolite-based tanning with retanning using Retanal (B and E), aluminium-based tanning with copolyamide finishing (D), titanium-based tanning with metal-free retanning (S) and phenolic-based tanning with metal-free retanning (CN, SN, CB and SB). The different processing methods are expected to provide a comprehensive understanding of how chemical composition and finishing techniques influence the degradation properties and environmental impact of the final leather products.

### 2.2. Leather Physicochemical Characterisation

The physicochemical properties of the leather samples were analysed, including the heavy metal content, total carbon (TC), total nitrogen (TN), and the C/N ratio, in order to obtain the necessary information to prepare an adequate initial mixture for composting (10–40 C/N) and calculate the maximum theoretical amount of CO_2_ [15]. The pH was analysed in a 1:10 (*w*/*v*) sample/water solution. The TC and TN were determined via dry combustion at 1020 °C in an automatic microanalyzer (Thermo Finnigan Flash 1112 Series, Waltham, MA, USA). The concentrations of several metals and metalloids (As, Cd, Cr, Cu, Mg, Mn, Ni, Pb, Se and Zn) were determined by means of inductively coupled plasma mass spectrometry (7800 ICP-MS, Agilent, Santa Clara, CA, USA) was used according to the CPSC-CH-E1002-08.03 method for metal content determination and the specifications of ISO standards [16].

To gain insight into finished leather biodegradation—particularly potential differences between the grain and flesh sides—pristine and treated samples were analysed using several infrared-based spectroscopy techniques and mercury porosimetry. A Bruker Analitik IFS 66 FTIR spectrometer (Ettlingen, Germany) equipped with a DTGS KBr detector and a Golden Gate Single Reflection Diamond ATR accessory (incident angle of 45°) was employed to measure the infrared absorption spectra from 500 to 4.000 cm^−1^ using 64 scans and a 4 cm^−1^ resolution. Additionally, all the samples were analysed by means of an 8700 LDIR Chemical Imaging System (Agilent Technologies, Santa Clara, CA, USA) equipped with a quantum cascade laser light source and a mercury cadmium telluride detector. While this instrument operates from 975 to 1800 cm^−1^, only two wavenumbers—associated with collagen (amide I band, 1635 cm^−1^) and leather tanning agents (1110 cm^−1^)—were specifically monitored at a resolution of 0.5 cm^−1^ to study leather decomposition. For each sample, a 10 × 10 µm area was measured, combining the spectrum from both wavenumbers. An Autopore IV 9500 Micrometrics mercury porosimeter (Micromeritics Instrument Corporation, Norcross, GA, USA) was employed for sample pore structure characterisation in the pore radius interval of 0.002–200 μm. To this end, and prior to the analysis, specimens were cut into regular samples of approximately 1 cm^3^ and were dried at 60 °C for 24 h.

### 2.3. Biodegradation Studies

#### 2.3.1. Compostability (ISO 14855-1:2012 Standard)

The composting experiment was conducted at the Laboratory of Biotechnology of the Footwear Technology Centre INESCOP (Elda, Alicante, Spain), following the controlled laboratory composting conditions according to the ISO 14855-1:2012 standard [12]. The compost system provided by ECHO Instruments (Slovenske Konjce, Slovenia) was run at 58 °C during the assay up to 100 days. The assay parameters, the composition of the bioreactors and the percentage of biodegradation (%) were determined as Vico et al. [10] described previously. The main characteristics of the mature compost inoculum and the operational conditions of the ISO 14855-1:2012 assay, including reactor mass, sample mass, aeration flow and measurement frequency, are provided in Table S1. A laboratory-scale composting process tailored to the materials under study was developed using cellulose (Sigma-Aldrich^®^, St. Louis, MI, USA) as a positive reference control substance.

#### 2.3.2. Biodegradation Liquid Phase (ISO 20136:2020 Standard)

Similar to the composting assays, the leather biodegradation assay was performed at the Laboratory of Biotechnology of the Footwear Technology Centre INESCOP (Elda, Alicante) following the ISO 20136:2020 standard [14]. The inoculum consisted of wastewater collected from the municipal wastewaters treatment plant in the local area of Elda (Alicante) (38°27′6.92″ N, 0°48′6.98″ W) and wastewater from a tannery biological tank in Segorbe (Castellón, Spain, 39°51′11.33″ N, 0°28′33.26″ W). Pure collagen from bovine Achilles tendon (Sigma-Aldrich^®^, St. Louis, MO, USA) was used as a positive control (control sample). The assay was run for approximately 800 h (33 days). A total of 0.18 g of each sample was mechanically disintegrated into small fibrous shavings using a MÜLLER laboratory blade mill, model LAB 500.5 (Francisco Muñoz Irles, Petrer, Spain), for 1–3 min per sample and transferred into Erlenmeyer flasks containing minimal salts medium and the inoculum. The material obtained was not a fine powder but small leather threads; therefore, no additional sieving step was performed. No refrigeration or specific thermal control was used, as the process did not generate noticeable heating. The total organic carbon content of the tested material was determined by elemental analysis. The material obtained was not a fine powder but consisted of small leather threads; no additional sieving step was performed. This allows the theoretical maximum quantity of carbon dioxide evolution to be calculated as biodegradation. All calculations were performed on a dry-mass basis according to the equations specified in ISO 20136:2020. For both assays, biodegradation (%) was calculated as the net cumulative CO_2_ evolved from the test material, after blank correction, divided by the theoretical maximum CO_2_ calculated from the total carbon content of the dry sample, multiplied by 100.

#### 2.3.3. Statistical Analysis

To compare the performance of the two standardised biodegradation assays (ISO 14855-1:2012 and ISO 20136:2020) this study analysed the behaviour of eight polymeric leather samples under each protocol, evaluating the degree of similarity in their response profiles. To this end, seven parameters were analysed: (1) the final biodegradation value; (2–4) the percentage of biodegradation reached when 1/3, 1/2, and 2/3 of the total test duration—expressed in days—had elapsed for each of the two standardised tests; and (5–7) the time required for each sample to reach 25%, 50%, and 75% of its maximum biodegradation, calculated once the biodegradation plateau had been reached. Positive controls were not included in the statistical analysis due to their distinct nature compared to the leather samples.

All seven indicators were assigned equal statistical weight. This choice was made because the objective of the multivariate analysis was to describe the overall biodegradation trajectory rather than to prioritise a single phase of the process a priori. Since the leather samples differed in tanning chemistry, retanning/fatliquoring systems and finishing treatments, different materials could plausibly exhibit distinct degradation patterns, with some differences emerging early in the assay and others becoming evident only at later stages or at the endpoint. Therefore, assigning unequal weights without independent prior evidence would have introduced an arbitrary assumption about which degradation phase was most relevant. Equal weighting was thus retained as a transparent exploratory approach.

To explore underlying data structures and reveal potential grouping patterns, hierarchical clustering was performed using squared Euclidean distance and Ward’s method to assess sample similarity and define exploratory groups [17]. Given the limited sample size, this analysis was used strictly as a descriptive tool to summarise global patterns across the seven biodegradation indicators; therefore, the resulting cluster structures should not be interpreted as confirmatory evidence. The optimal number of clusters was determined by jointly inspecting the Elbow criterion on the within-cluster sum of squares and the maximum average Silhouette width, thereby balancing compactness and separation. As a complementary visual check, t-distributed stochastic neighbour embedding (t-SNE) plots [18] were generated (Supplementary Materials) to assess whether the low-dimensional visual arrangement was consistent with the dendrogram-based grouping, rather than to define clusters or the resulting partition.

Accordingly, emphasis was placed on consistency of patterns across indicators and assays rather than on formal inference. The analysis was independently replicated for each assay to ensure methodological consistency and robust comparison across ISO protocols. Hierarchical clustering was performed using JASP, version 0.19.3 (https://jasp-stats.org/).

### 2.4. Ecotoxicity Studies

The maturity of the compost obtained was assessed through its chemical properties and an ecotoxicological evaluation using two complementary assays, the Zucconi germination index [19] and OECD 208 protocol (Terrestrial Plant Test: Seedling Emergence and Seedling Growth Test) [20] using Raphanus sativus L. to determine the relative growth of plants (RGrP, %) [10]. This combined approach provided a comprehensive assessment of final compost quality and potential environmental impact. For the liquid-phase biodegradation assay (ISO 20136:2020), however, phytotoxicity of the aqueous extracts generated during leather degradation was only assessed with the Zucconi index, as it was impossible to apply the OECD 208 protocol for liquid samples.

### 2.5. Graphical Abstract Preparation

The graphical abstract was developed using a combination of original graphical elements prepared by the INESCOP Design Department and AI-generated visual elements. OpenAI’s image generation and editing tool through ChatGPT (specific image-model version not displayed in the interface) was used to generate some illustrative elements and to perform the final composition and layout of the graphical abstract. The tool was not used to generate, process, analyse, or interpret experimental data. All scientific content, terminology, labels, and visual outputs were reviewed and validated by the authors.

## 3. Results and Discussion

Over the years, different methodologies have been applied to evaluate how different finishing techniques might affect leather biodegradation. Although these methodologies share the objective of improving the sustainability of leather, their scope and methodological approaches differ substantially, which makes comparisons difficult. Furthermore, it should be considered that the samples tested in these studies receive diverse chemical treatments (e.g., tanning and retanning), which presents an additional challenge for comparative analysis. Unlike previous studies [21,22], the present work represents the first direct comparison of two biodegradation methodologies applied to leather (ISO 14855-1:2012 vs. ISO 20136:2020), thus providing novel insights into the consistency and complementarity of these approaches in assessing the end-of-life behaviour of eco-friendly leathers. To this end, a set of eight commercial leather samples treated with various eco-friendly finishes were investigated.

### 3.1. Characterisation of Pristine Leather Samples

**Figure 1:**
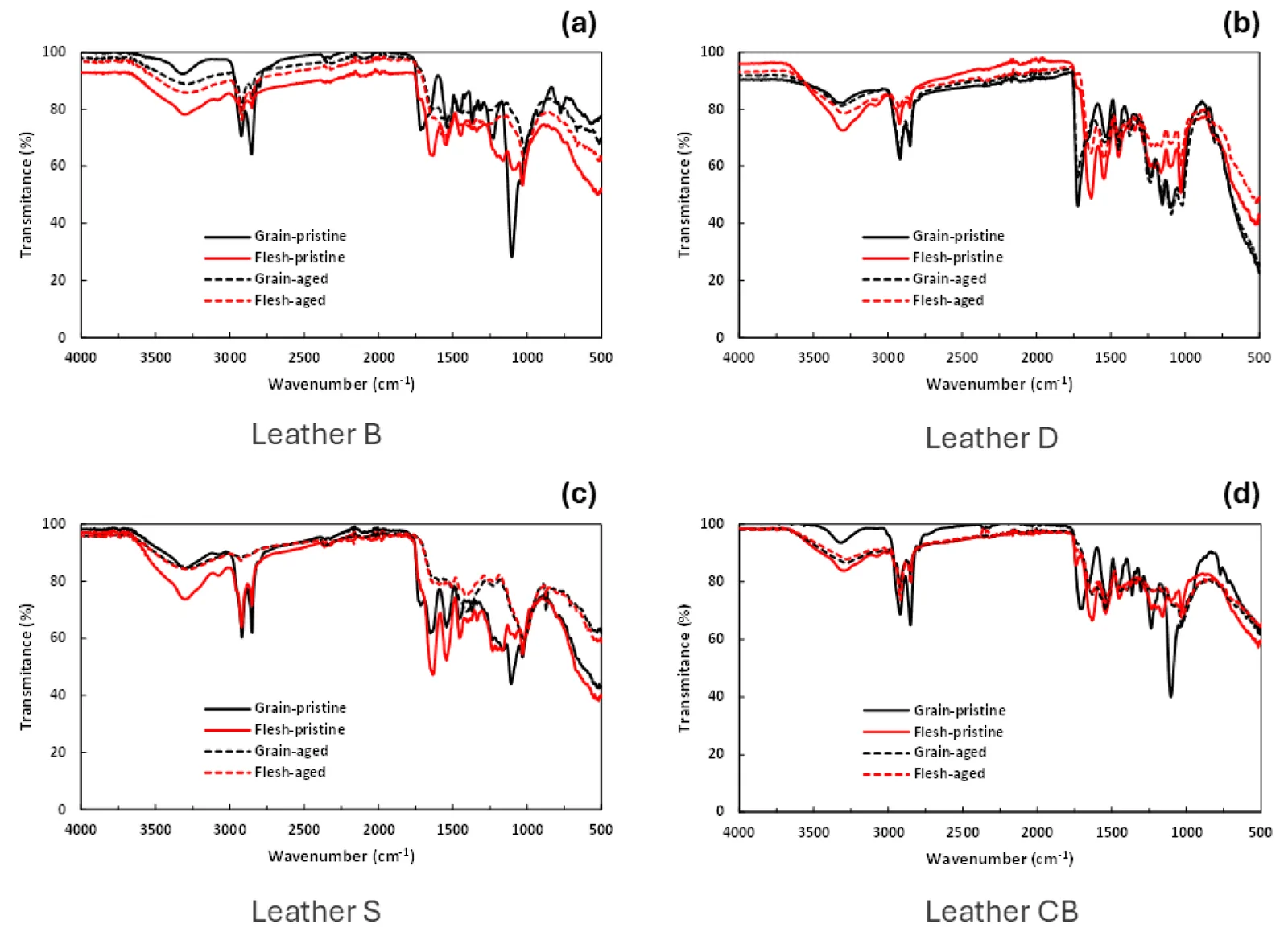
Infrared spectra of pristine and aged chromium-free tanned leather samples. Spectra are shown for representative tanning systems: (**a**) leather B (zeolite/aluminosilicate-based), (**b**) leather D (aluminium-based), (**c**) leather S (titanium-based) and (**d**) leather CB (phenolic-based). Black and red solid lines indicate pristine grain and flesh sides, respectively, whereas black and red dashed lines indicate aged grain and flesh sides, respectively.previously reported in the literature for bovine leathers, confirming the uniformity of the protein-based collagen matrix across tanning systems [25,26]. Nevertheless, the overall C/N ratio remains relatively low (i.e., 3.46–5.84), which may represent a challenge factor for composting performance under controlled laboratory conditions since it is significantly below the optimal range of 10–20 recommended for laboratory-scale biodegradation assays. The low C/N ratios can be attributed to the inherently high nitrogen content of leather, primarily derived from its collagenous protein matrix. Such an imbalance may hinder microbial activity by creating an excess of nitrogen relative to carbon, potentially slowing the rate of biodegradation or affecting microbial community dynamics.

Initially, all samples in this work were physicochemically characterised using various analytical techniques to fully understand the modifications derived by composting and biodegradation treatments, as well as potential differences among them. First, the eco-friendly tanned leathers were analysed via ATR-FTIR spectroscopy, which provides valuable molecular-level insights into structural changes resulting from the tanning and retanning processes. Because these treatments might differ between the grain and flesh sides, both sides were analysed independently. Figure 1 shows the IR spectra for four pristine leather samples, which are representative of the different tanning processes applied, based on information provided by the manufacturer (Table 1). Tanning treatments applied were based on the use of zeolites (sample B), aluminium (sample D), titanium (S), and phenolic compounds (CB). For each sample, the IR spectrum of both the grain and flesh sides was analysed because the tanning and retanning agent treatments applied to them are not strictly equivalent. The data shown in Figure 1 corresponds to the average of five independent measurements taken through the sample. As expected, pristine samples showed a mixture of the characteristic bands of collagen and those related to the chemicals used for tanning and retanning. In fact, the former are more intense on the flesh side, which is, a priori, less chemically treated than the grain side. The main collagen-related bands observed were: (i) the Amide A (stretching vibrations of the amide N–H bonds) at 3300 cm^−1^; (ii) Amide B (resonance overtone of the amide II vibration) at 3080 cm^−1^; (iii) Amide I ((mostly C=O stretching) 1637 cm^−1^; (iv) Amide II (C-N stretching and N–H bending) 1544 cm^−1^; and (v) Amide III (NH bending, CN stretching vibration and small contributions from both CO in plane bending and CC stretching vibration) 1237 cm^−1^ [23]. Irrespective of the sample nature, the IR spectrum showed several bands potentially associated with leather finishing procedures such as: (i) asymmetric and symmetric stretching of −CH_2_ and −CH_3_ group at 2920 and 2850 cm^−1^ usually associated with waxes and oils employed to impart softness and flexibility to leather [24]; (ii) stretching of C=O at 1719 cm^−1^, indicating vegetable tanning procedures, since vegetable tanning involves complex mixtures of polyphenols (retans) that may contain such functional groups; and (iii) carbon–oxygen bond stretching at 1110 cm^−1^ which could also be related to tanning agents. A priori, the observed differences in the intensity of these bands suggest a greater degree of alteration in the leather’s internal structure. Similar findings were found for the remaining samples investigated (i.e., E, CN, SB, and SN) (Figure S1, Supplementary Materials).

Additionally, a more in-depth chemical characterisation was conducted by measuring the following parameters (Tables S2 and S3): (i) TC content (%); (ii) maximum theoretical CO_2_ production. This information allows to determine the final biodegradation percentage; (iii) TN content (%); (iv) C/N ratio; and (v) elemental composition (i.e., As, Cd, Cr, Cu, Hg, Mo, Ni, Pb, Se, Zn). Despite the differences in tanning technologies and retanning processes, all samples exhibited comparable and typical elemental compositions in terms of carbon and nitrogen content. The values observed, with carbon ranging between 41 and 49% and nitrogen between 8 and 13%, are consistent with those reported in the literature.

In terms of elemental composition, the concentration of metalloids and heavy metals in the pristine leather samples was below the maximum reference limits established for compostability studies (Table S2) in the EN 13432:2000 standard [16]. While most samples showed significant levels of Cr and Zn, no clear relationship was found between the levels of these two elements. The levels of Cr ranged from 8.57 mg kg^−1^ (sample D) to 47.66 mg kg^−1^ (sample S), whereas the levels of Zn were between <10 mg kg^−1^ (sample CN) and 138.2 mg kg^−1^ (sample E).

### 3.2. Biodegradation Assays

#### 3.2.1. Compostability Based on the ISO 14855-1:2012 Standard

All leather samples were analysed for compostability at a laboratory scale according to the ISO 14855-1:2012 standard. The compostability was calculated based on the cumulative aerobic biodegradation, measured as the percentage of C converted to CO_2_ relative to the initial C content. This value represents how much of the material’s organic C has been biodegraded and converted to CO_2_ by microorganisms over a specific period. While 100% biodegradation is the ideal, values can exceed this due to the variability in the composition of both the test material and the inoculum (mature compost employed), as well as the inherent measurement uncertainty of the method [27,28]. For the sake of comparison, cellulose was used as a positive control to validate the test conditions and ensure the compostability test was functioning properly, as cellulose is explicitly recommended for this purpose in ISO 14855-1:2012.

**Figure 2:**
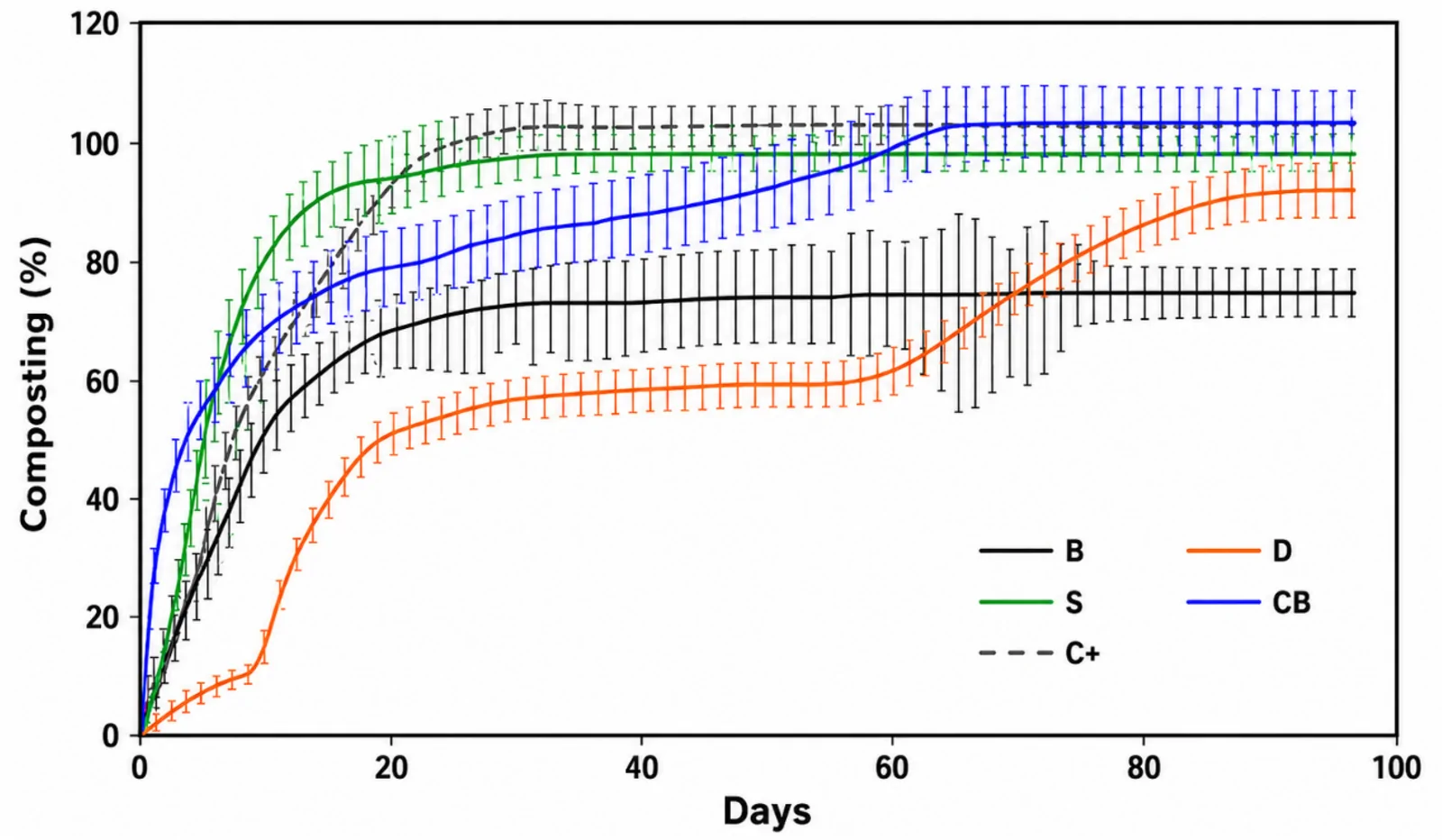
Composting kinetic curves of chromium-free tanned leathers according to ISO 14855-1:2012. The *x*-axis represents test time (days), and the *y*-axis represents cumulative aerobic biodegradation/composting (%), calculated from CO_2_ evolution relative to the theoretical maximum CO_2_. Curves represent mean values, and error bars indicate standard deviation (SD) (*n* = 3). C+ denotes the cellulose positive control.

Figure 2 shows the composting kinetic curves of leather samples B, D, S, and CB, which are representative of the different eco-friendly tanning procedures applied to the samples investigated. In general, as expected from the Cr-free tanning strategies used, leather compostability was significant in all cases, above 70%. Samples S and CB achieved complete biodegradation similar to that of the cellulose reference, in agreement with the work of Reetz et al. [21].

Interestingly, beyond the final values, the compostability kinetics revealed marked differences among the samples. For instance, sample S displayed a steep and rapid increase in CO_2_ evolution, reaching over 70% compostability within the first days and achieving complete degradation in less than 20 days. Interestingly, though sample B was not fully compostable (76.16%), the kinetic curve was similar to that shown for sample S. In contrast, composting of sample CB was more significant during the first 5 days than for the remaining samples and then showed a steadier progression until complete compostability after approximately 70 days. Finally, the compostability of sample D reached a plateau after 20 days (65%) but then continued to decompose, achieving a second plateau at 90.2%. This latter behaviour was also shown by samples E, SB, CN and SN (Figure S2).

These differences in kinetics suggest that the tanning and retanning agents strongly influence the onset and rate of microbial activity. Leathers treated with titanium (sample S) or phenolic agents (e.g., sample CB and CN) supported a faster microbial attack, whereas zeolite- and aluminium-based systems (samples B and E) showed a delayed onset and lower degradation rate under our test conditions. Thus, while most samples ultimately achieved compostability values above 90%, the degradation pathways and speeds were heterogeneous, reflecting, on the one hand, the distinct chemical compositions and structural features imparted by each tanning strategy, but also the different collaborative degradation strategies, through the production of collagenolytic enzymes, employed by the various microorganisms present in the compost [8,10,29].

To further investigate leather compostability, aged samples were analysed by ATR-FTIR to assess molecular-level changes compared to pristine ones, focusing on the differences between the grain and flesh sides since tanning and retanning treatments applied to them are not equivalent (Figure 1 and Figure S1). Irrespective of the sample, the leathers transformed into a black amorphous mass and lost their original square shape (25 × 25 mm) making it difficult to distinguish between the grain and flesh sides. Furthermore, because the mass of the leather remaining after the compostability assay was minimal due to high biodegradability, samples from an intermediate time point (11–14 days)—corresponding to less than 40% compostability—were specifically analysed instead. As expected, ATR-FTIR analysis confirmed the progressive biodegradation of both the grain and flesh layers of the leather throughout the composting process. In the former case, the most prominent spectral change was the drastic reduction in the aliphatic stretching peaks at 2920 and 2850 cm^−1^, alongside the significant attenuation of the carbon–oxygen bond stretching at 1110 cm^−1^, both of which are associated with the degradation or leaching of synthetic fatliquors and tanning agents. Concurrently, for both sides of the leather, a noticeable flattening and loss of resolution were observed within the amide I and II regions (1700–1500 cm^−1^). This spectral alteration provides direct evidence of the enzymatic cleavage and structural destabilisation of the collagen triple helix matrix during organic decomposition. All these aforementioned changes were particularly significant for leather S (final compostability 102.6%) since all the relevant spectral features mostly disappeared, and there was no difference between the grain and flesh sides. For samples B and D (final compostability <90%), however, leather- and tanning-related bands were still recognisable, and it was still possible to differentiate between the two sample sides.

**Figure 3:**
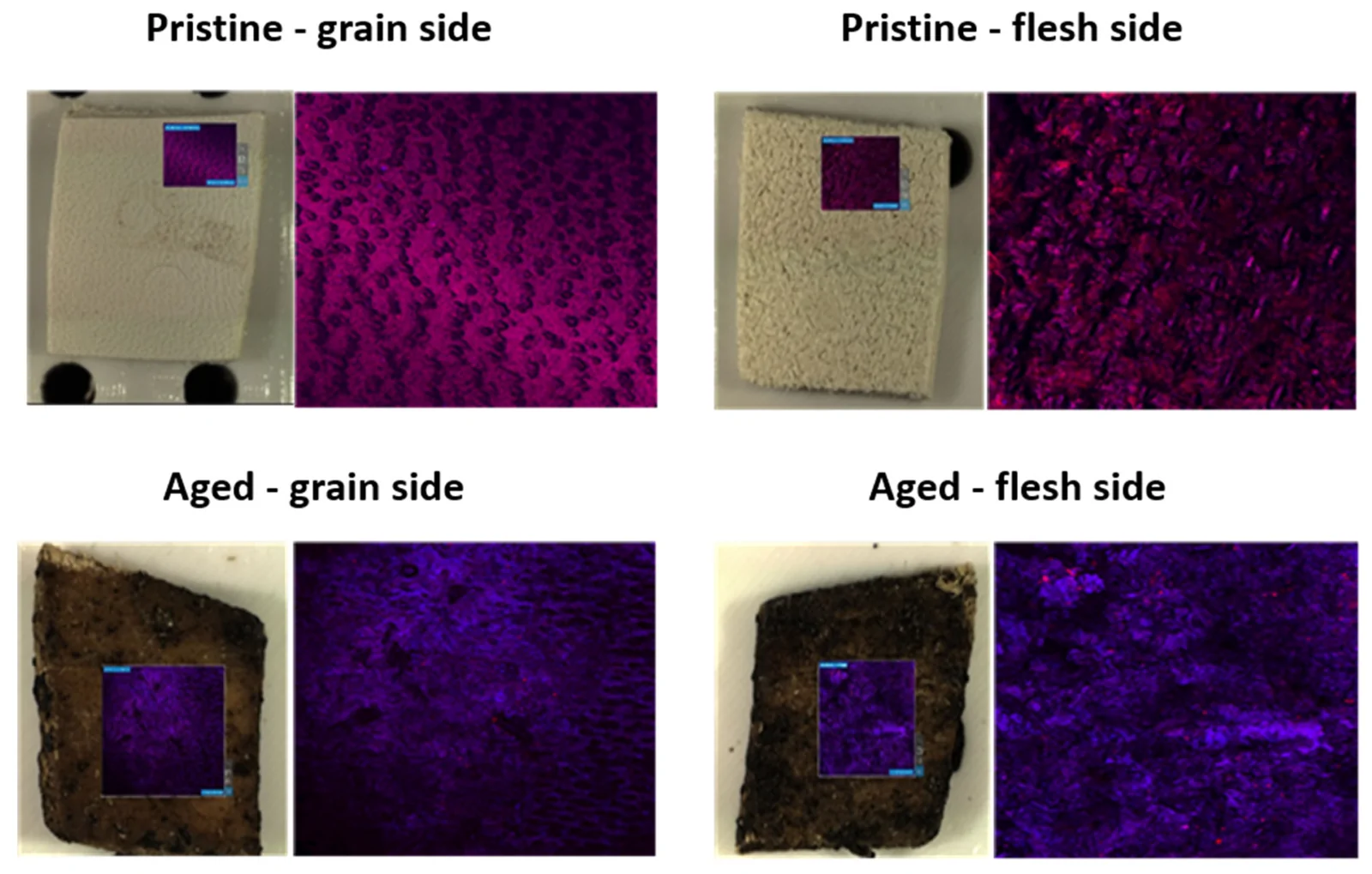
LDIR images of pristine and aged leather sample CB during compostability testing according to the ISO 14855-1:2012 standard. For each subset, the photograph is paired with the corresponding 10 × 10 μm LDIR chemical map. Blue regions indicate the collagen-associated signal (amide I band, 1635 cm^−1^), red regions indicate the tanning-agent-associated signal (1110 cm^−1^), and purple regions indicate overlapping contributions from both signals.

**Figure 4:**
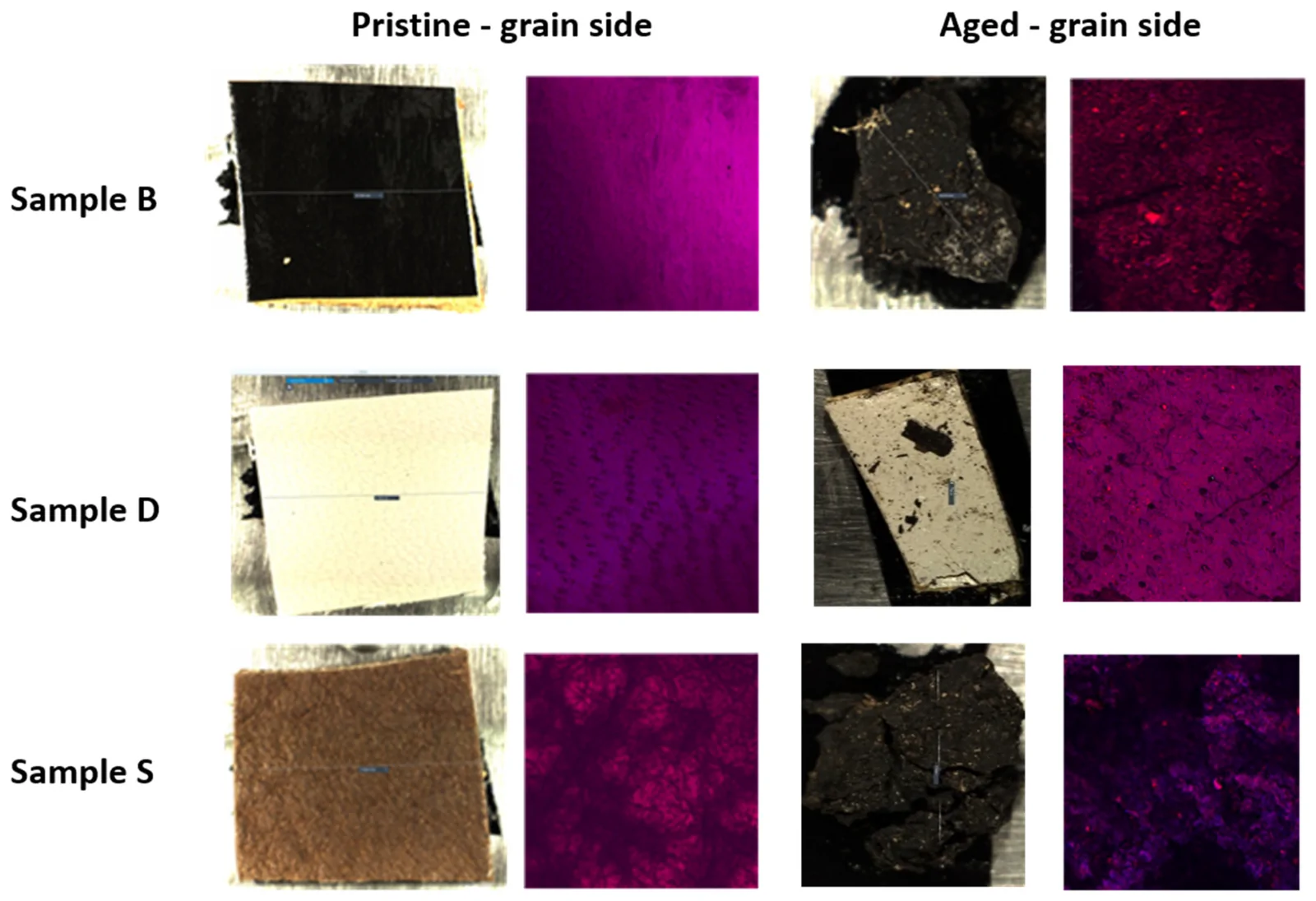
LDIR images of pristine and aged grain sides for leather samples B, D and S. For each subset, the visual image is paired with the corresponding 10 × 10 μm LDIR chemical map. Blue regions indicate the collagen-associated signal (amide I band, 1635 cm^−1^), red regions indicate the tanning-agent-associated signal (1110 cm^−1^), and purple regions indicate overlapping contributions from both signals.

Alongside ATR-FTIR characterisation, aged leather samples were analysed using laser direct infrared (LDIR) imageing spectroscopy. LDIR is a cutting-edge technique utilised in microplastic identification [30], pharmaceutical analysis [31], and tissue examination but its application to leather remains unexplored. Unlike ATR-FTIR, which suffers from limited spatial resolution, this method enables detailed examination of solid surfaces to better elucidate material changes during biodegradation. The instrument operates by focusing the laser beam onto a microscopic spot on the sample, measuring the absorbed IR light in the 900–1800 cm^−1^ range. This data is then used to generate a chemical image, with a pixel’s location corresponding to the IR absorbance at a specific wavenumber. In this work, wavenumbers associated with collagen (amide I band, 1635 cm^−1^) are mapped in blue, whereas tanning agents (carbon–oxygen bond stretching, 1110 cm^−1^) regions are mapped in red. Overlapping regions containing both wavenumbers appear purple. Figure 3 shows the LDIR images for both grain and flesh sides of sample CB with and without ageing. The images on the left correspond to the visual appearance of the samples to the naked eye, whereas the images on the right represent the chemical maps combining the two aforementioned wavelengths over a 10 × 10 µm area. As expected from tanning and retanning treatment procedures in leather samples, both grain and flesh sides appear purple, but colour intensity was slightly higher on the grain side. Following ageing, however, the samples display a more heterogeneous surface dominated by a blue colour, indicating that the tanning and retanning agents initially present on the leather surface had been partially degraded or removed during the biodegradation process. Nonetheless, isolated red spots remain visible in certain regions. These results clearly demonstrate that chemically treated regions with eco-friendly tanning and retanning agents are biodegradable, thereby facilitating material composting. This behaviour aligns well with the high degree of compostability exhibited by this sample. Similar findings were also observed for sample S (Figure 4), but in this case, the number of red spots was more significant and no clear blue spot regions were noticed. Regarding samples B and D (Figure 4), the images are predominantly characterised by purple and red hues, which correlates with the lower compostability rates exhibited by these samples. Based on these findings, the tanning and retanning agents used in these latter samples apparently hamper composting to a higher degree during the early stages of the biodegradation process. This might explain the lower composting rate shown by these samples. The results for the remaining samples studied were in line with these findings.

Finally, 312. Table S4 shows the interparticulate and intraparticulate porosity for all samples, calculated from their intrusion curves. Interparticulate porosity refers to the void space between individual particles, while intraparticulate porosity refers to the void space within the particles themselves. According to Table S4, pristine samples exhibit significantly different inter- and intraparticle porosities, reflecting variations in composition and tanning treatments. Upon ageing, both porosity types decreased significantly across all samples, with average reductions of 40% for interparticle porosity and 60% for intraparticle porosity. Given that porosity does not increase over time, all the leather samples tested appear to undergo uniform, gradual degradation as a whole, irrespective of the tanning and retanning agents employed.

#### 3.2.2. Biodegradability in Liquid Phase Based on the ISO 20136:2020 Standard

**Figure 5:**
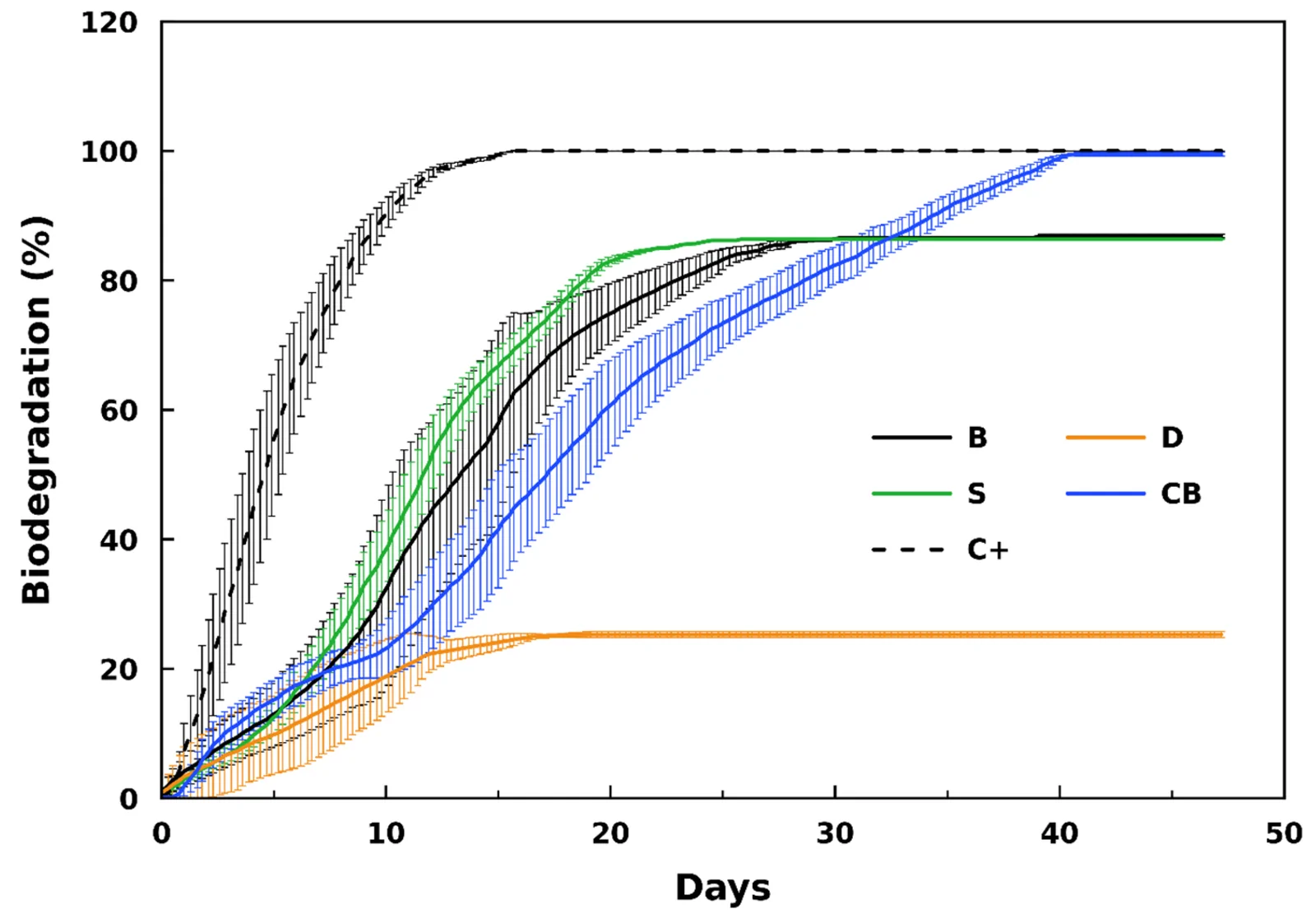
Biodegradation kinetic curves of chromium-free tanned leathers according to ISO 20136:2020. The *x*-axis represents test time (days), and the *y*-axis represents cumulative aerobic biodegradation (%), calculated from CO_2_ evolution relative to the theoretical maximum CO_2_. Curves represent mean values, and error bars indicate standard deviation (SD) (*n* = 2). C+ denotes the collagen positive control.

The biodegradability of leather samples in the liquid phase was evaluated according to the ISO 20136:2020 standard. Unlike the ISO 14855-1:2012 standard, originally developed for plastics and later adapted to leather, this method was specifically designed to assess leather degradation in an aqueous medium. Biodegradability was calculated as the cumulative aerobic conversion of the organic C present in the samples into CO_2_ relative to their initial C content. An inoculum composed of a mixture of wastewater from a tannery biological tank and a municipal biological tank provided the microbial consortium for the assay, while pure collagen was used as the positive control to validate test conditions. In this standard, biodegradation is determined by comparing the CO_2_ released during the experimental period with the theoretical maximum CO_2_ evolution, estimated from the TC (%) of each sample. The 28-day criterion applies only to validation of the positive control, whereas samples were monitored until reaching a plateau. Overall, except for leather D, all the analysed samples exhibited similar biodegradation rates and of above 82% (Figure 5). Interestingly, biodegradation kinetics for the reference (collagen) was significantly faster than those for the eco-friendly tanned leathers. Thus, the former was completely biodegraded within 10–15 days whereas the leathers achieved only 20–30% biodegradation during the same period. In contrast, sample D showed the lowest biodegradability at 25.3% (<20% after 50 days), suggesting significant resistance to decomposition under the test conditions and thus remaining virtually inert. The origin of this behaviour cannot be assigned to a single factor from the present dataset. It should instead be interpreted as the response of the complete commercial finished leather system under aqueous ISO 20136:2020 conditions. In this assay, mechanical disintegration into fibrous shavings increases contact between the medium and the whole material, including the collagen matrix, the aluminium-based tanning system, the co-polyamide finishing layer and any other formulation components. However, greater surface exposure does not necessarily imply greater enzymatic accessibility of the collagen fraction, because collagen hydration, swelling and crosslinking can influence transport and enzymatic action [8,32]. The low biodegradation of sample D may therefore reflect a combination of collagen stabilisation, limited swelling or enzyme access, the contribution of the co-polyamide/plastic-film finish and other proprietary formulation features, rather than direct inhibition of the inoculum by leached aluminium alone. To further evaluate whether soluble aluminium release could account for this behaviour, an additional liquid-phase verification assay was performed using collagen supplemented with Al(III), added as Al_2_(SO_4_)_3_·18H_2_O, at D-, B- and E-equivalent aluminium doses. These doses were calculated from the total aluminium contents of the corresponding leathers and assuming a conservative 50% release from the 0.18 g sample mass used in the ISO 20136:2020 assay (Figure S4).

Soluble aluminium partially decreased collagen mineralisation relative to the positive control. However, even the highest aluminium dose tested did not reproduce the markedly low biodegradation observed for sample D. These results indicate that aluminium leaching may contribute to slowing mineralisation in the liquid-phase assay, but it is not sufficient on its own to explain the behaviour of sample D. This supports the interpretation of sample D as a complete finished-leather architecture, in which aluminium-based tanning chemistry, collagen stabilisation, swelling, enzyme accessibility, the co-polyamide/plastic-film finish and other proprietary formulation components may jointly influence the response under ISO 20136:2020 conditions.

Unlike the compostability study, it was not feasible to investigate the ISO 20136:2020 residues in detail by IR techniques and porosimetry because the tested material was mechanically disintegrated into small fibrous shavings and dispersed in the aqueous medium, preventing a comparable surface or porosity assessment of coherent leather pieces.

#### 3.2.3. Compostability and Biodegradation Kinetic Curves Comparison

The analysis of the biodegradation curves shows that, under ISO 20136:2020 conditions, most leather samples reached their maximum biodegradation on a shorter timescale than under ISO 14855-1:2012. This difference should not be attributed solely to particle size. ISO 20136:2020 combines an aqueous, well-mixed medium, a liquid microbial consortium, ambient temperature, a shorter assay duration and mechanically disintegrated fibrous shavings. By contrast, ISO 14855-1:2012 exposes larger leather pieces to a thermophilic compost matrix with a more heterogeneous solid environment, longer incubation, greater microbial diversity and a higher buffering capacity. Therefore, the two standards impose different biological and physicochemical constraints on the same finished leather systems, which explains why some materials can converge in their endpoint values whereas others diverge markedly.

To gain insight into leather degradation using both approaches as well as to evaluate the influence of the tanning and retanning procedure, an in-depth statistical analysis was carried using the kinetic curves previously shown for both approaches (Figure 2 and Figure 5, Figures S2 and S3). To this end, seven analytical indicators were established to evaluate the statistical behaviour of the samples throughout the biodegradation process through both ISO 14855-1:2012 and ISO 20136:2020: (i) biodegradation percentages at 1/3, 1/2 and 2/3 of the total test duration (in days, as defined for each standard); (ii) the time required for each sample to reach 25%, 50% and 75% of its maximum biodegradation, calculated once the biodegradation plateau had been reached; and (iii) the final biodegradation value measured at the end of each test. Equal statistical weight was assigned to all seven indicators. The use of seven equally weighted indicators allowed the biodegradation process to be considered as a trajectory rather than as a single endpoint. This is relevant because some samples shared the same broad tanning chemistry but differed in finishing or retanning/fatliquoring treatments. For example, B and E were both zeolite/aluminosilicate-based leathers, whereas CN, SN, CB and SB belonged to phenolic-based systems, but their finishing systems were not identical. Consequently, samples with the same broad tanning family could still differ in the rate, timing and final extent of biodegradation. For this reason, the multivariate analysis was used to summarise the kinetic evolution of the curves (Figures S5–S8).

**Figure 6:**
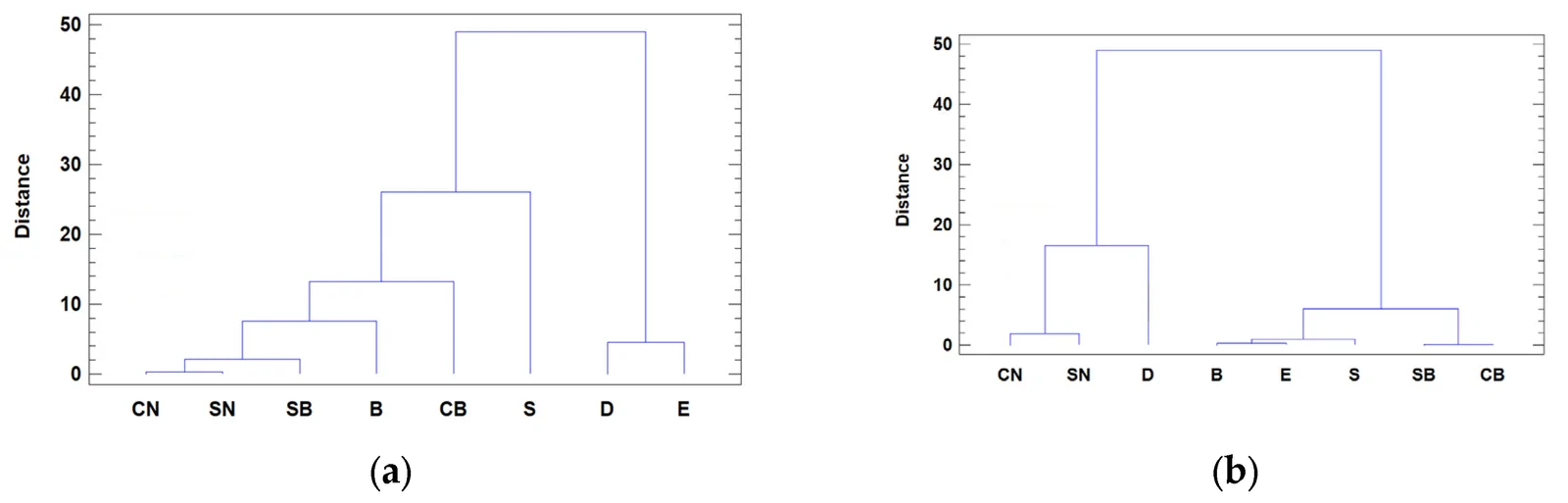
Dendrogram of (**a**) ISO 14855-1:2012 and (**b**) ISO 20136:2020 tests.

Figure 6 displays the dendrograms obtained for both standards; complementary t-SNE plots and cluster indicator summaries are provided in the Supplementary Materials (Figures S5–S9). The results reveal three clearly differentiated clusters for ISO 14855-1:2012 (Figure 6a): (i) Cluster 1, comprising samples B, CB, CN, SB, and SN; (ii) Cluster 2, consisting exclusively of sample S; and (iii) Cluster 3, which includes samples D and E. Among the indicators investigated, both biodegradation percentages at 1/2 and 2/3 of the total test duration exhibited the highest discriminatory power, effectively differentiating the three identified profiles. Specifically, these variables provided clear separation between Cluster 3 (low profile) and Cluster 1 (medium-high profile). Furthermore, they distinctly isolated Cluster 2, which was characterised by exceptionally high values. Biodegradation percentage at 1/3 and the time required to reach 50% and 75% of its maximum biodegradation showed high discrimination, particularly in distinguishing Cluster 1 (positive values) from Cluster 3 (negative values). For Cluster 2, these variables exhibited a mixed pattern—showing positive values for biodegradation percentage at 1/3 and the time required to reach 75% of its maximum biodegradation but negative values for the time required to reach 50% of its maximum biodegradation. The remaining parameters investigated (maximum biodegradation, minimum threshold and time required to reach 25%) were less relevant for discrimination. Cluster 1 corresponded to a medium-high biodegradation profile with broadly consistent progression through the assay. Cluster 2 contained only sample S, reflecting a distinct behaviour relative to the other leathers. Cluster 3 grouped samples D and E, defining a separate profile in which biodegradation evolved differently over time compared with Cluster 1. This pattern indicates that, under composting conditions, mid- and late-stage mineralization dynamics are particularly informative, which is consistent with the heterogeneous nature of compost matrices and the influence of colonisation, diffusion and progressive enzymatic accessibility of collagen during the process [32].

As regards the ISO 20136:2020 assay (Figure 6b), three different clusters were obtained: (i) Cluster 1, comprising samples CN and SN; (ii) Cluster 2, consisting of samples B, E, S, CB and SB; and (iii) Cluster 3, which exclusively includes sample D. Biodegradation percentages at 1/3 and 1/2 exhibit the highest discriminatory capacity, providing a clear separation between Cluster 2 (positive values) and Cluster 1 (strongly negative values). Additionally, they facilitate the differentiation of Cluster 3 (values near zero) from both Cluster 1 and Cluster 2. Biodegradation percentage at 2/3 and the time required to reach 25% of its maximum biodegradation clearly differentiated Cluster 2 (positive values) from Cluster 1 (negative values). Since Cluster 3 also registers negative values, these variables are highly effective at isolating Cluster 2 from the rest of the groups (Cluster 1 and Cluster 3); however, they offer lower resolution when distinguishing between Cluster 1 and Cluster 3. Cluster 2 included samples B, E, S, CB and SB, representing a second profile characterised by a distinct early-to-mid evolution relative to Cluster 1. Cluster 3 consisted exclusively of sample D, which was statistically isolated and aligned with its markedly reduced biodegradation in this assay. Taken together, these outcomes suggest that, in ISO 20136:2020, early-to-mid biodegradation development is particularly informative for defining sample similarity. This pattern is coherent with the higher contact between the material and the aqueous medium created by mechanical disintegration and agitation. ISO 20136:2020 appears particularly sensitive to how each finished leather system behaves when its collagen matrix, tanning chemistry and finishing layer are exposed to a liquid microbial environment [27,33].

The dendrogram-based exploratory clustering results show that each ISO protocol highlights different kinetic phases and, therefore, different constraints. In ISO 14855-1:2012, clusters are mainly discriminated by mid-to-late mineralisation (1/2 and 2/3 of the test duration), consistent with heterogeneous compost where colonisation fronts, diffusion and progressive enzymatic accessibility drive the trajectory [32]. In ISO 20136:2020, separation is dominated by early-to-mid development (1/3 and 1/2), coherent with maximised accessibility (grinding/agitation) and greater sensitivity to inhibition by soluble/leachable species.

**Figure 7:**
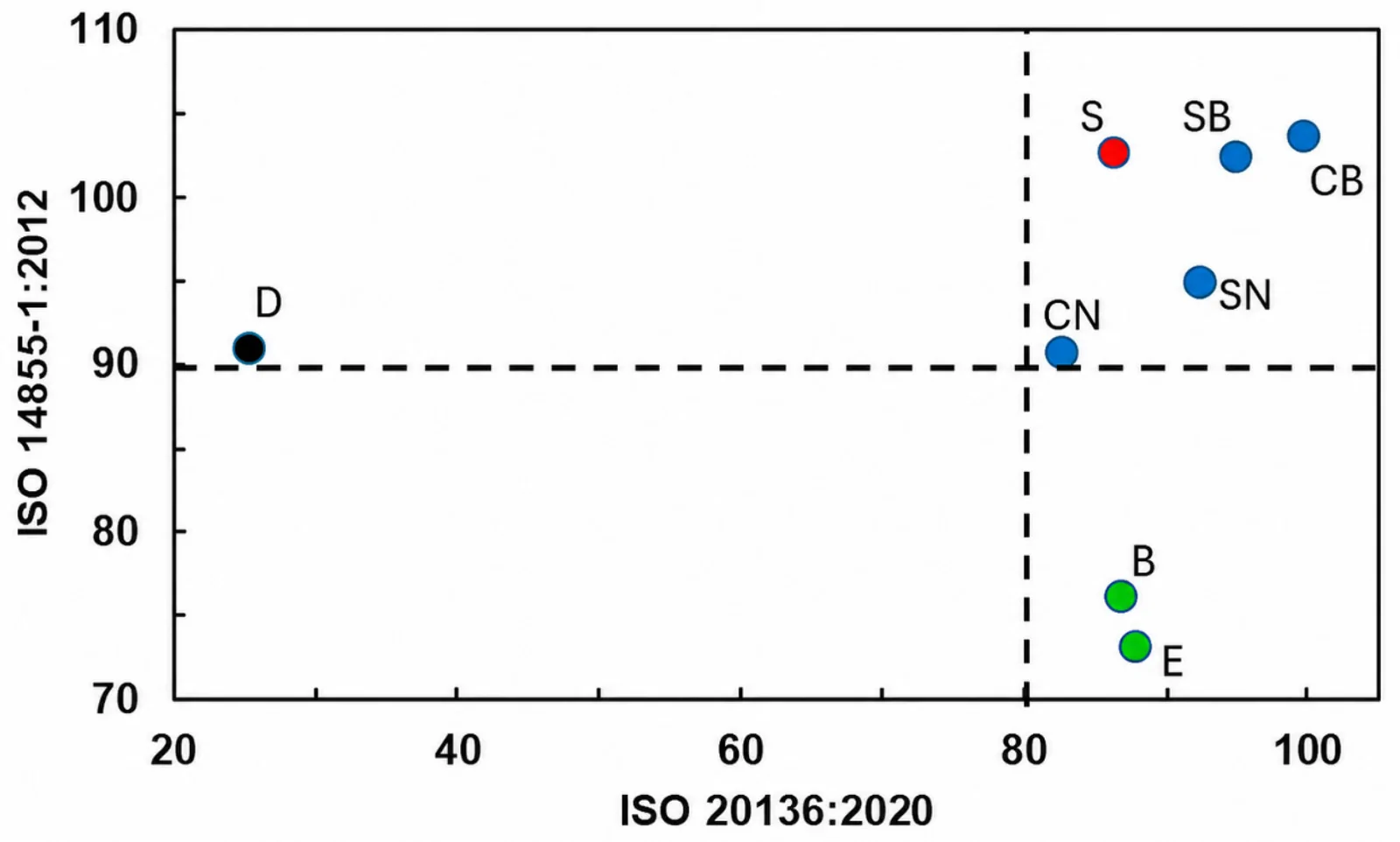
Scatter plot comparing endpoint biodegradation (%) under ISO 20136:2020 (*x*-axis) and ISO 14855-1:2012 (*y*-axis) for the leather samples. Marker colours identify the main tanning system: black circle = aluminium-based, blue circles = phenolic-based, red circle = titanium-based and green circles = zeolite/aluminosilicate-based. Dashed reference lines indicate 80% biodegradation under ISO 20136:2020 and 90% biodegradation under ISO 14855-1:2012.

Overall, the two approaches showed broadly consistent endpoint classifications for several samples, particularly phenolic and titanium-tanned leathers, while also revealing relevant method-dependent divergences for zeolite- and aluminium-based systems (Figure 7). Absolute endpoint differences between ISO 14855-1:2012 and ISO 20136:2020 ranged from 0.32 to 14.77 percentage points for all samples except D, which showed the largest divergence (65.65 percentage points).

Leathers tanned with phenolic and titanium agents exhibited high final biodegradation values in both assays; results for ISO 14855-1:2012 exceeded 90%, while ISO 20136:2020 results were above 80%, in some cases even approaching or surpassing 90%. This agrees with collagen stabilisation governed by non-covalent interactions and metal–ligand coordination [34,35,36], while retaining swelling and water uptake sufficient to sustain transport and enzymatic action [32,37]. Polyphenol crosslinking (hydrogen bonding, hydrophobic and π–π interactions) can increase stability without necessarily suppressing biodegradation in well-hydrated matrices with diverse enzymatic consortia [24,38], consistent with burial studies showing progressive loosening of collagen–tannin matrices and detanning/gelatinisation under microbial action [24,39]. In contrast, zeolite/aluminosilicate-tanned leather showed a different type of method-dependent divergence: high aqueous biodegradability aligns with well-mixed liquid reports [21,40,41], whereas compost endpoints for B and E are lower (70–80%). In these systems, aluminium is mainly associated with aluminosilicate/zeolite structures and should not be considered chemically equivalent to soluble aluminium salts or to aluminium coordinated within an aluminium-tanned collagen matrix. The lower compost endpoints for B and E are therefore more plausibly related to mineral-rich microstructures and inorganic load affecting wetting, swelling, colonisation and diffusion in a heterogeneous compost matrix. The current data do not allow the effect of Zn or any other single component to be isolated. These results illustrate how ISO 14855-1:2012 reveals compost-specific accessibility limitations that may be masked under ISO 20136:2020 conditions.

Finally, the most marked divergence between the two standards was observed for sample D, which showed only 25% biodegradation in ISO 20136:2020 but exceeded 90% in ISO 14855-1:2012. This behaviour should not be interpreted solely on the basis of a single isolated factor. The aluminium present in finished leather may exist in different chemical environments, and aluminium involved in collagen stabilisation is not directly comparable with aluminium contained in zeolite/aluminosilicate systems or with soluble aluminium salts. For sample D, the available formulation information also does not allow the effect of the aluminium-based tanning chemistry to be separated from that of the co-polyamide finishing layer and other proprietary components. The divergence is therefore best interpreted as the response of the complete finished leather architecture to two different test environments. In ISO 20136:2020, the disintegrated material is exposed to a liquid consortium at 23 °C for a shorter period; limited swelling, restricted enzyme access to collagen, or the presence of less biodegradable finishing domains may strongly influence the measured mineralisation. In ISO 14855-1:2012, the longer thermophilic composting assay provides a more diverse microbial community, a solid organic matrix and greater buffering capacity, which can support progressive colonisation and mineralisation of leather pieces over time [42,43,44]. In this context, the 80% value used in Figure 7 for ISO 20136:2020 should not be interpreted as a universal or normative threshold. In the present dataset, it was used only as an internal descriptive reference point, since all samples except D exceeded this level in the liquid-phase assay, whereas D showed a clearly divergent low biodegradation value. Given the limited number of samples and the absence of an independent classification reference, no formal cut-off optimisation was attempted. Nevertheless, the observed separation suggests that an ISO 20136:2020 endpoint around 80% may be considered a preliminary reference for future validation studies involving larger and more chemically diverse leather datasets.

Overall, using both standards is informative: ISO 14855-1:2012 emphasises compost-driven colonisation, diffusion and community buffering, whereas ISO 20136:2020 highlights liquid-phase exposure of the disintegrated finished leather and the effective accessibility of its collagen fraction. Across assays, clustering supports tanning/retanning chemistry and finished-leather architecture as primary drivers of biodegradation behaviour; however, the individual contribution of aluminium tanning, finishing chemistry and other formulation components cannot be isolated for sample D from the present data.

### 3.3. Phytotoxicity and Ecotoxicity

While ISO 14855-1:2012 and ISO 20136:2020 provide valuable information on the biodegradation behaviour of leather under controlled composting and aqueous conditions, respectively, biodegradation alone does not necessarily ensure the environmental safety of the resulting matrices. A material may undergo extensive mineralisation while still releasing soluble compounds, salts, metals, tanning or retanning residues, finishing additives, or intermediate metabolites capable of affecting seed germination, root elongation, seedling emergence, or early plant growth. Therefore, ecotoxicological assessment is an essential complementary endpoint in any circular or compostability-oriented evaluation scheme, as it helps to verify that the degradation process does not generate byproducts with adverse biological effects [16,20,45]. This is particularly relevant for finished leather, whose environmental behaviour depends not only on the collagen matrix but also on the chemical nature of the tanning, retanning, and finishing systems applied during manufacture.

In this study, phytotoxicity was first assessed by means of the Germination Index (GI), using two types of aqueous matrices: the liquid extract obtained from the compost produced in the ISO 14855-1:2012 procedure and the liquid medium resulting from leather degradation under ISO 20136:2020 conditions (Table 2). The GI is a combined biological indicator that integrates relative seed germination and relative root elongation with respect to the control [19,46]. This assay is especially useful as a rapid screening tool for detecting water-soluble phytotoxic compounds, since early germination and radicle development are highly sensitive to inhibitory substances, salinity, ammonium, organic acids, and other soluble compounds commonly associated with immature or unsuitable organic amendments [45,47]. In the present study, all GI values were above 90%, exceeding the 80% threshold commonly used to indicate absence of relevant phytotoxicity in compost-derived materials [47,48]. These results suggest that neither the compost extracts nor the aqueous biodegradation media generated from the tested eco-friendly finished leathers exerted relevant inhibitory effects on seed germination or root elongation. This observation is consistent with previous results reported for composted finished leathers [10]. In the specific case of sample D, the absence of phytotoxicity in the ISO 20136:2020 liquid medium is noteworthy, considering its low biodegradation rate under this standard, and suggests that the reduced microbial mineralisation observed in the aqueous assay cannot be directly translated into phytotoxicity towards seeds.

The ecotoxicity assay, adapted from OECD guideline 208, was applied only to the composted leather materials obtained under ISO 14855-1:2012, since this endpoint requires a soil-like solid matrix and is therefore not suitable for the liquid media generated under ISO 20136:2020. Unlike the GI assay, which mainly reflects short-term effects of soluble compounds on germination and radicle elongation, the OECD 208-based growth test provides a more integrative assessment of plant response in a solid substrate, closer to the potential environmental or agronomic use of the compost. In this assay, the relative growth of plants (RGrP, %) was determined in a 50:50 (*w*/*w*) mixture of compost and standard commercial substrate and compared with plants grown in 100% commercial substrate. The acceptance criterion for absence of relevant ecotoxicity was set at RGrP ≥ 90%.

Except for samples B and CB, all composted materials met this criterion, indicating no relevant growth inhibition under the tested conditions. The standard deviations associated with RGrP were low, ranging from 1.84 to 3.76 percentage points, supporting the descriptive repeatability of the growth response observed among replicates. Samples B and CB showed RGrP values of 86.74 ± 2.41% and 85.14 ± 2.69%, respectively, slightly below the non-toxicity threshold and therefore consistent with a marginal inhibitory effect rather than severe ecotoxicity. By contrast, samples D and CN yielded RGrP values clearly above 100% (120.50 ± 3.74% and 110.98 ± 3.44%, respectively), suggesting a possible growth-promoting response under the experimental conditions.

This stimulatory effect should be interpreted cautiously, since the final nutrient composition, nitrogen speciation, electrical conductivity, salinity, free amino acid content, and peptide profile of the composts were not specifically determined in this study. Nevertheless, the response is biologically plausible. Leather is a collagen-rich proteinaceous material, and the tested pristine leathers showed high nitrogen contents and low C/N ratios, reflecting the nitrogen-rich nature of the collagen matrix. During composting, partial transformation of this organic nitrogen may contribute to nutrient availability or to the formation of bioactive organic nitrogen compounds. In this regard, protein hydrolysates and peptide/amino acid-containing products have been widely described as plant biostimulants capable of improving nutrient uptake, nitrogen metabolism, and plant growth, depending on their composition, dose, and origin [49,50,51]. Therefore, the RGrP values above 100% observed for some composted leather samples may reflect not only the absence of toxicity but also a favourable interaction between the compost matrix and early plant development. However, this interpretation remains hypothetical and should be confirmed in future work through a more detailed agronomic characterisation of the composts.

Overall, the combined use of GI and OECD 208-based growth assessment provides complementary information on the environmental safety of the biodegradation outputs. The GI assay is particularly sensitive to soluble phytotoxic compounds released into aqueous extracts, whereas the plant growth test evaluates the biological response in a solid substrate containing compost. This distinction is important for interpreting sample D: although this sample showed low biodegradability under ISO 20136:2020, suggesting limited microbial accessibility or possible inhibition of the liquid-phase inoculum, its composted material showed no ecotoxic effect in the OECD 208-based assay. The two endpoints measure different biological responses and should not be conflated. Low biodegradation in the liquid-phase assay reflects limited effective mineralisation of the complete finished leather system under those test conditions, but it does not necessarily imply phytotoxicity or general toxicity. In liquid assays, the disintegrated material and any soluble components are in direct contact with the microbial consortium, whereas composting matrices are more heterogeneous and may buffer, dilute, immobilise or biologically transform compounds while supporting progressive colonisation over a longer thermophilic incubation.

An important limitation in the current standardisation framework is the absence of a dedicated, leather-specific compostability standard integrating biodegradation, composting performance, chemical safety, and ecotoxicological endpoints in a harmonised way. Existing compostability schemes, such as EN 13432:2000, were developed for packaging and plastic materials and include ecotoxicity as a key requirement for final compost quality, but they are not fully adapted to the specific composition, collagen-based structure, and processing chemistry of finished leather. In this context, the combined application of ISO 14855-1:2012, ISO 20136:2020, GI, and OECD 208-based plant growth testing provides a more robust framework for assessing the circular and environmental suitability of finished leather biodegradation products. These results indicate a generally low phytotoxic and ecotoxic impact of the composts obtained from the tested eco-friendly finished leathers, while also highlighting the need for future studies to include nutrient speciation and plant-growth-promoting indicators to better understand the mechanisms underlying the stimulatory responses observed in some samples.

Compared with other polymer degradation and recycling routes, including chemical depolymerisation/devulcanisation and thermal conversion, the ISO-based biological approaches used here provide a milder and more environmentally oriented assessment of end-of-life behaviour. Chemical and thermal routes can be advantageous for recovering purified polymers, monomers, chemical intermediates, fuels or syngas from polymer wastes, including recalcitrant systems such as polyolefins and elastomers [52,53,54]. However, these routes often involve high-temperature processing, catalysts, high energy input, product upgrading or downstream treatment to improve selectivity and manage contaminants [53,54], and, unlike the framework used here, they do not directly assess the biological response of the resulting degradation matrix unless coupled with dedicated ecotoxicological tests. Future work should therefore combine biodegradation kinetics, chemical safety, compost quality and larger chemically diverse leather datasets to define robust benchmarks and support the development of leather-specific compostability and biodegradation protocols.

## 4. Conclusions

This work provides a comparative, standards-based assessment of the biodegradation of finished leathers, integrating composting (ISO 14855-1:2012) and aqueous-medium testing (ISO 20136:2020) with complementary physicochemical characterisation and ecotoxicity/phytotoxicity endpoints. We found that combining these ISO methods offers clearer insights than either alone; while results converged for several materials, the dual approach revealed key divergences that a single test environment would have missed. In this dataset, phenolic- and titanium-tanned finished leathers consistently exhibited high biodegradation across both assays. The aluminium-based sample behaved quite differently, showing high compost mineralization but low aqueous mineralization. This divergence should be interpreted as a method-specific response of the complete finished leather system rather than as evidence of a single inhibitory mechanism.

Finally, within this limited dataset, a biodegradation level of approximately ≥80% in ISO 20136:2020 separated sample D from the remaining leathers and was broadly aligned with samples showing high biodegradation in ISO 14855-1:2012. This value is used here only as a descriptive, study-specific reference point and should not be interpreted as a normative or universal classification threshold, particularly because chemically distinct finished leather systems can respond differently depending on the assay matrix, inoculum and exposure conditions. Its potential use as a preliminary benchmark would require validation with larger and more chemically diverse leather datasets.

The phytotoxicity and ecotoxicity tests were equally encouraging, indicating low overall environmental impact of biodegradation byproducts under the tested conditions. Our findings provide a practical framework for waste-management and end-of-life evaluation of finished leathers in which biodegradation performance is interpreted jointly with chemical/structural information and (eco)toxicity endpoints, thereby strengthening evidence for decision-making and material screening.

## Figures and Tables

**Table 1:** Chemical families and functional roles of the tanning, retanning/fatliquoring and finishing systems used for the eco-friendly tanned leathers tested.

Sample	Tanning	Retanning/Fatliquoring	Finish
B	Chrome-free zeolite/aluminosilicate-based system; organic acid fixation; hydrophobic/fatliquoring auxiliaries.	Synthetic retanning agents; phospholipid/fatliquoring auxiliaries; dye auxiliaries.	Pigment/dye; mineral fillers; natural wax; bio-based polyurethane dispersion; bio-based acrylic binder; polyurethane rheology modifier; polyaziridine crosslinker.
E	Chrome-free zeolite/aluminosilicate-based system; organic acid fixation; hydrophobic/fatliquoring auxiliaries.	Synthetic retanning agents; phospholipid/fatliquoring auxiliaries; dye auxiliaries.	Pigment/dye; mineral fillers including silica-containing matte system; synthetic wax; polyurethane dispersion; acrylic binder; polyurethane rheology modifier; polyaziridine crosslinker.
D	Chrome-free aluminium-based system (probable aluminium chemistry according to supplier/process records).	Not disclosed in the available supplier/process records.	Copolyamide-based finishing product supported on a plastic film.
S	Chrome-free titanium-based system.	Not disclosed in the available supplier/process records.	Aromatic and aliphatic polyurethane aqueous dispersions; acrylic resin blend; modified protein/casein dispersion; wax/silicone aqueous emulsion.
CB	Chrome-free phenolic-based system; lecithin/oil-based fatliquoring component and phenolic derivative reported in the tanning stage.	Inorganic aluminium-containing salt; organic polysaccharide/phenolic retanning agent; lecithin-based fatliquors; vegetable oils; milk protein; sodium polyacrylate; surfactant auxiliaries.	White pigment; polyacrylate/acrylic polymer binder; water-based polyurethane.
CN	Chrome-free phenolic/syntan-type system.	Synthetic retanning agents; vegetable tannin component (tara-type); surfactant/fatliquoring auxiliaries; fixation auxiliaries.	Dye/pigment system; wax component; polyurethane and acrylic binder/dispersions; auxiliary film-forming components.
SB	Chrome-free phenolic-based system; lecithin/oil-based fatliquoring component and phenolic derivative reported in the tanning stage.	Inorganic aluminium-containing salt; organic polysaccharide/phenolic retanning agent; lecithin-based fatliquors; vegetable oils; milk protein; sodium polyacrylate; surfactant auxiliaries.	White pigment application reported; no further finishing/topcoat system was reported.
SN	Chrome-free phenolic/syntan-type system.	Synthetic retanning agents; vegetable tannin component (tara-type); surfactant/fatliquoring auxiliaries; fixation auxiliaries.	Dye/pigment reported; no finishing/topcoat system was reported.

Notes Due to confidentiality agreements signed with the participating companies, commercial product names, supplier identities and exact proprietary formulations cannot be disclosed. Chemical families and technological functions are therefore reported instead of commercial names. For sample D, the available information does not allow the effect of the aluminium-based tanning chemistry to be separated from the effect of the copolyamide finishing system.

**Table 2:** Phytotoxicity and ecotoxicity assays using liquid extract and compost after leather degradation.

Sample	Germination Index (GI, %)		Relative Plant Growth (RGrP, %)	Standard Deviation (SD)
	ISO 20136:2020	ISO 14855-1:2012	ISO 14855-1:2012	RGrP
B	115.05	98.71	86.74	2.4
D	102.58	90.09	120.50	3.7
E	90.65	105.59	92.42	3.8
S	114.00	102.98	94.32	2.7
CB	105.37	102.55	85.14	2.7
CN	117.02	96.16	110.98	3.4
SB	107.04	103.09	100.00	1.8
SN	110.23	95.95	102.00	3.4

Optimum values: Phytotoxicity above 80%; RGrP above 90%. Note: GI and RGrP values are expressed relative to their respective controls; therefore, values above 100% indicate a biological response higher than the control under the tested conditions and should not be interpreted as percentages of biodegradation or analytical errors.

## Data Availability

The original contributions presented in this study are included in the article and Supplementary Material. Further inquiries can be directed to the corresponding authors.

## Supplementary Materials

The following supporting information can be downloaded at: https://www.mdpi.com/article/10.3390/polym18192389/s1, Figure S1: Infrared spectra of pristine and aged chromium-free tanned leather samples; Figure S2: Composting kinetic curves of chromium-free tanned leathers according to ISO 14855-1:2012; Figure S3: Bio-degradation kinetic curves of chromium-free tanned leathers according to ISO 20136:2020; Figure S4: Effect of soluble aluminium on collagen biodegradation under ISO 20136:2020-like liquid-phase conditions; Figure S5: Distribution of the standardised bio-degradation indicators used in the clustering for ISO 14855-1:2012; Figure S6: Cluster means of the standardised indicators for ISO 14855-1:2012; Figure S7: Distribution of the standardised biodegradation indicators used in the clustering for ISO 20136:2020; Figure S8: Cluster means of the standardised indicators for ISO 20136:2020; Figure S9: Complementary t-SNE visualisation for (a) ISO 14855-1:2012 and (b) ISO 20136:2020; Table S1. Characteristics and chemical composition of the compost mature inoculum; Table S2. Characteristics and chemical composition of all the eco-friendly tanned leathers tested; Table S3. Heavy metal, metalloid, and fluorine content in the initial leather samples and their corresponding final composts. All results are expressed in mg kg-1 on a dry mass basis; Table S4. Interparticulate and intraparticulate porosity of pristine and treated leather samples; Table S5. Leather composting data from ISO 14855-1:2012 for statistical analysis and features (variables) used for ISO 14855-1:2012 statistical analysis; Table S6. Leather liquid-phase biodegradation data from ISO 20136:2020 for statistical analysis and features (variables) used for ISO 20136:2020 statistical analysis.
