# Supplementary material for: Biodegradability of Chromium-Free Finished Leather Through ISO 14855-1:2012 and ISO 20136:2020: A Multi-Faceted Analytical Approach
Source: Polymers (Basel). 2026 Sep 30;18(19):2389. doi: 10.3390/polym18192389 (PMC13667235; doi:10.3390/polym18192389)
Supplement: Supplementary file 1 [file polymers-18-02389-s001.zip › polymers-4581124-supplementary.pdf]

Supplementary Materials

Biodegradability of Chromium-Free Finished Leather Through ISO 14855-1:2012 and ISO 20136:2020: A Multi-Faceted Analytical Approach

Alberto Vico[1], Maria I. Maestre-López[1], Jose Miguel Carot-Sierra[2], Irene Lifante-Martinez[1], Elena Orgilés-Calpena[1], Guillermo Grindlay[3,*] and Marcelo Bertazzo[1,*].
1 Footwear Technological Institute (INESCOP), C/Alemania 102—Polígono Campo Alto, 03600 Elda, Spain
2 Department of Applied Statistics, Operational Research, and Quality, Universitat Politècnica de València, Camino de Vera s/n, 46022 Valencia, Spain
3 Department of Analytical Chemistry, Nutrition, and Food Sciences, University of Alicante, PO Box 99, 03080 Alicante, Spain
* Correspondence: guillermo.grindlay@ua.es (G.G.); mbertazzo@inescop.es (M.B.)

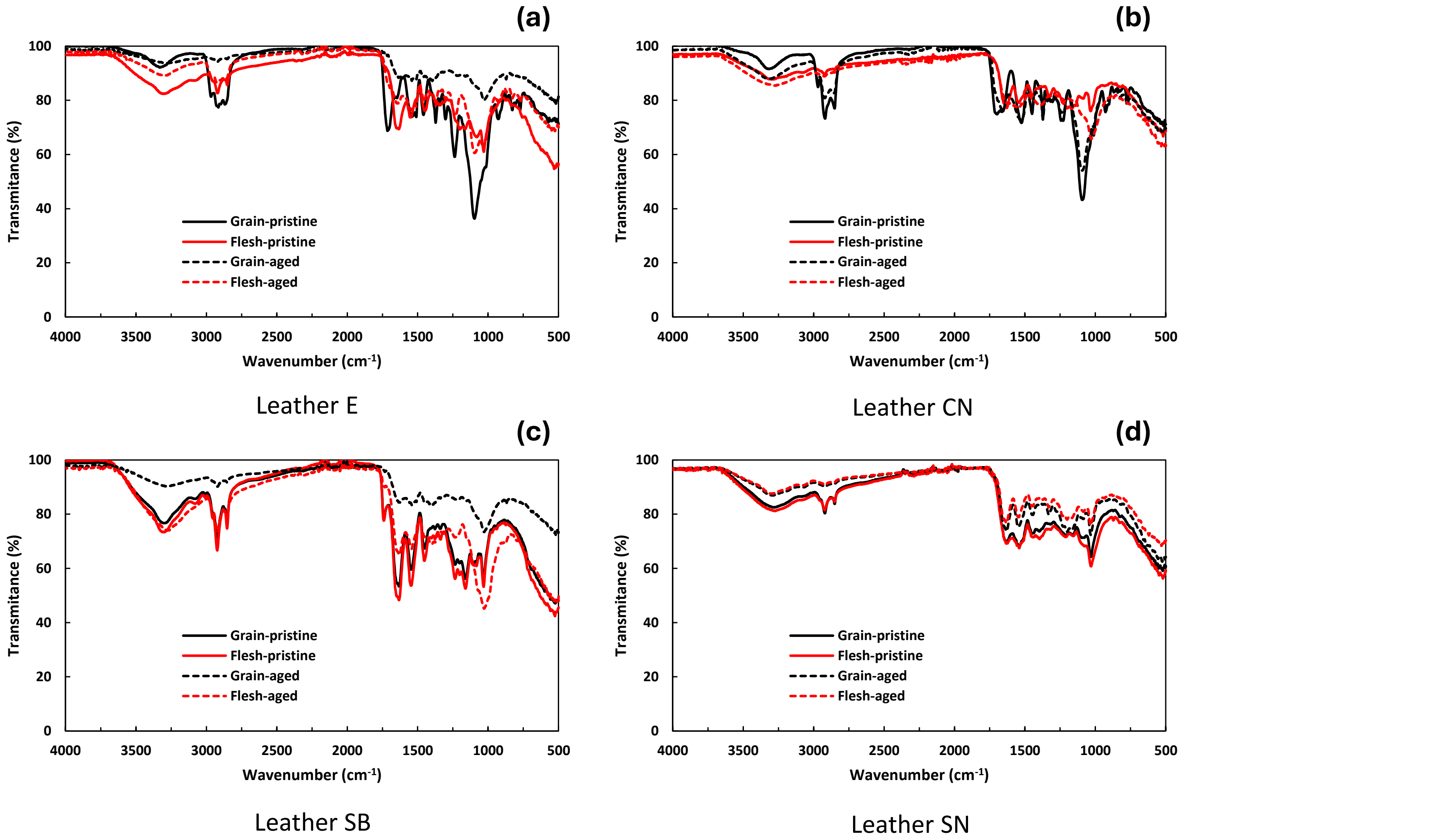

Figure S1. Infrared spectra of pristine and aged chromium-free tanned leather samples: (a) leather E, (b) leather CN, (c) leather SB and (d) leather SN. Black and red solid lines indicate pristine grain and flesh sides, respectively, whereas black and red dashed lines indicate aged grain and flesh sides, respectively.

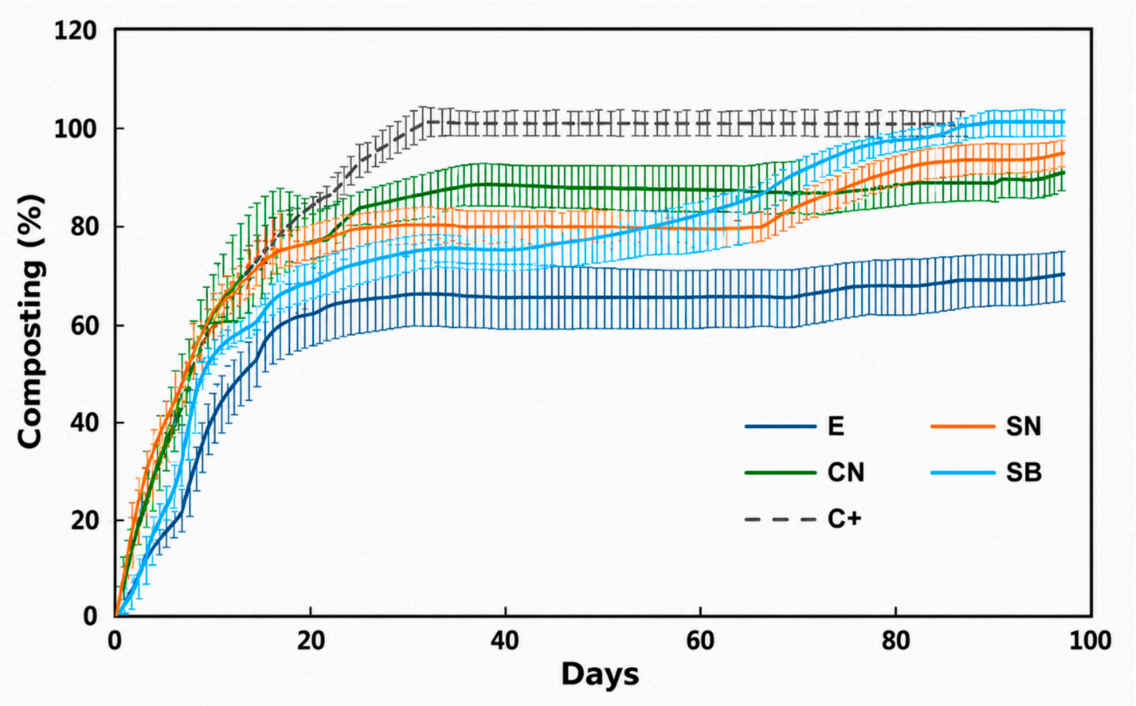

Figure S2. Composting kinetic curves of chromium-free tanned leathers according to ISO 14855-1:2012. The x-axis represents test time (days), and the y-axis represents cumulative aerobic biodegradation/composting (%), calculated from CO_2 evolution relative to the theoretical maximum CO_2. Curves represent mean values, and error bars indicate standard deviation (SD) (n = 3). C+ denotes the cellulose positive control.

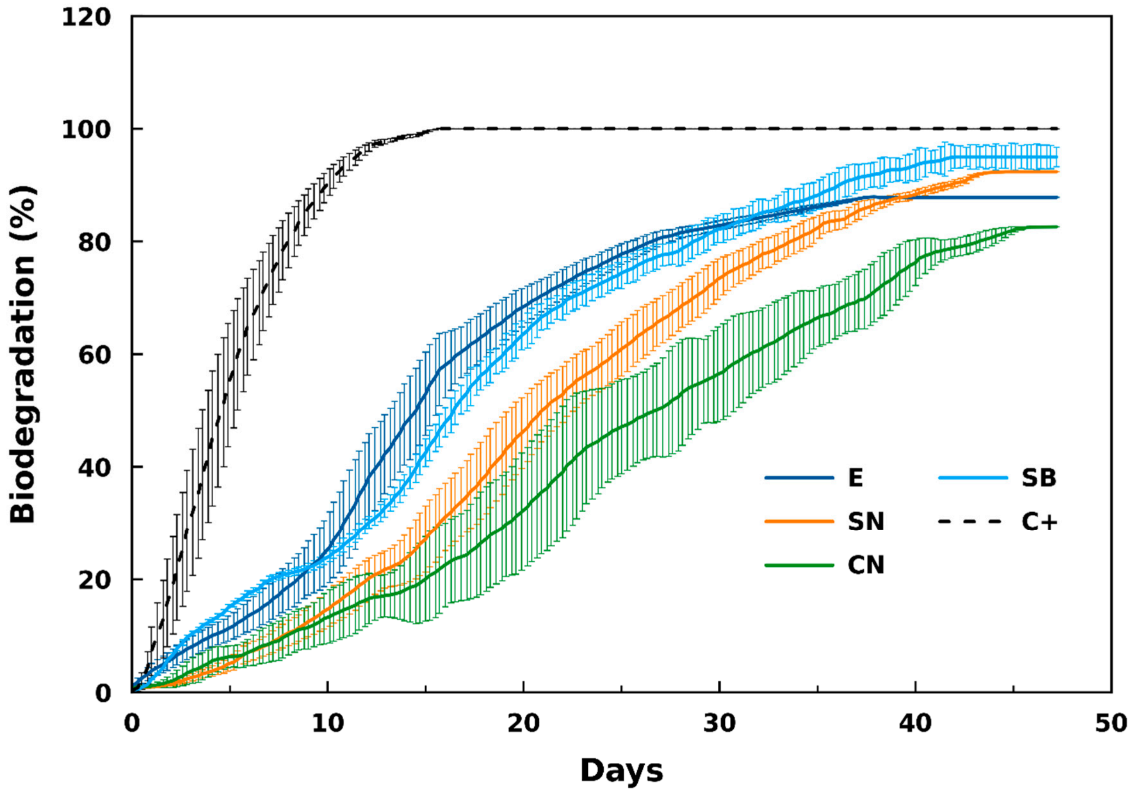

Figure S3. Biodegradation kinetic curves of chromium-free tanned leathers according to ISO 20136:2020. The x-axis represents test time (days), and the y-axis represents cumulative aerobic biodegradation (%), calculated from CO_2 evolution relative to the theoretical maximum CO_2. Curves represent mean values, and error bars indicate standard deviation (SD) (n = 2). C+ denotes the collagen positive control.

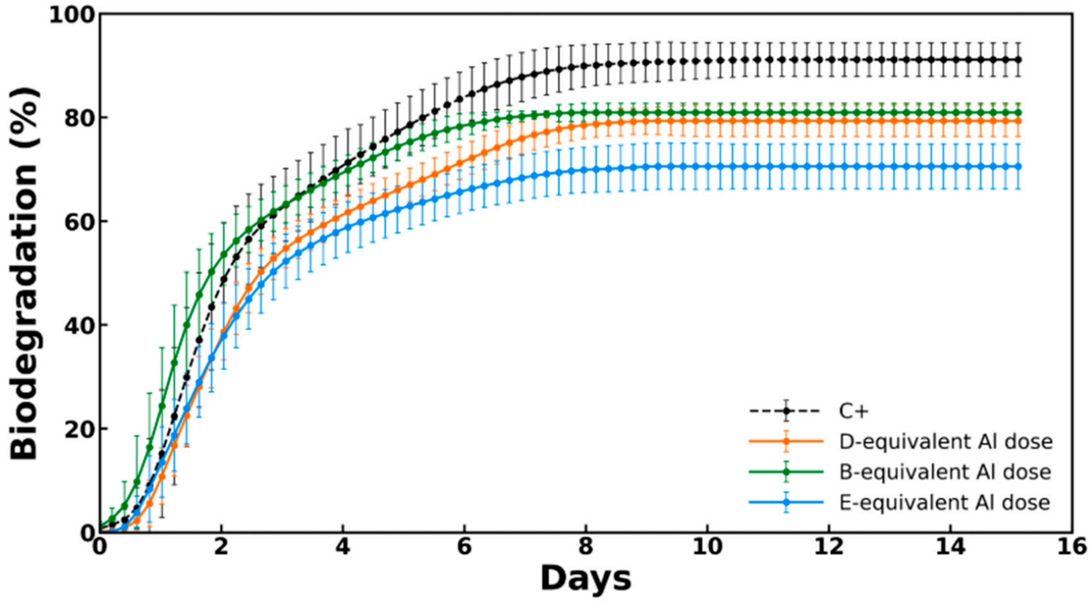

Figure S4. Effect of soluble aluminium on collagen biodegradation under ISO 20136:2020-like liquid-phase conditions. Aluminium was added as $Al_2(SO_4)_3 \cdot 18H_2O$ and expressed as elemental Al. The tested doses correspond to the theoretical release of 50% of the total aluminium present in 0.18 g of leather samples D, B and E, respectively. C+ corresponds to collagen without added aluminium.

Exploratory t-SNE visualisations

Supplementary Figures S5a and S5b show the t-SNE visualisations obtained from the seven standardised biodegradation indicators for ISO 14855-1:2012 and ISO 20136:2020, respectively. These plots are provided only as complementary exploratory visualisations and were not used to define the clusters discussed in the main text. Given the limited number of samples, t-SNE results should not be interpreted as confirmatory evidence. Nevertheless, the spatial arrangement of the samples was broadly consistent with the dendrogram-based interpretation. For ISO 14855-1:2012, sample S appeared separated from the remaining leathers, while samples D and E showed a distinct profile relative to the larger group comprising B, CB, CN, SB and SN. For ISO 20136:2020, sample D appeared clearly separated from the other samples, consistent with its markedly lower biodegradation in the liquid-phase assay. Thus, the t-SNE representation supports the exploratory grouping observed in the hierarchical clustering, while the dendrogram remains the primary basis for the discussion in the main manuscript.

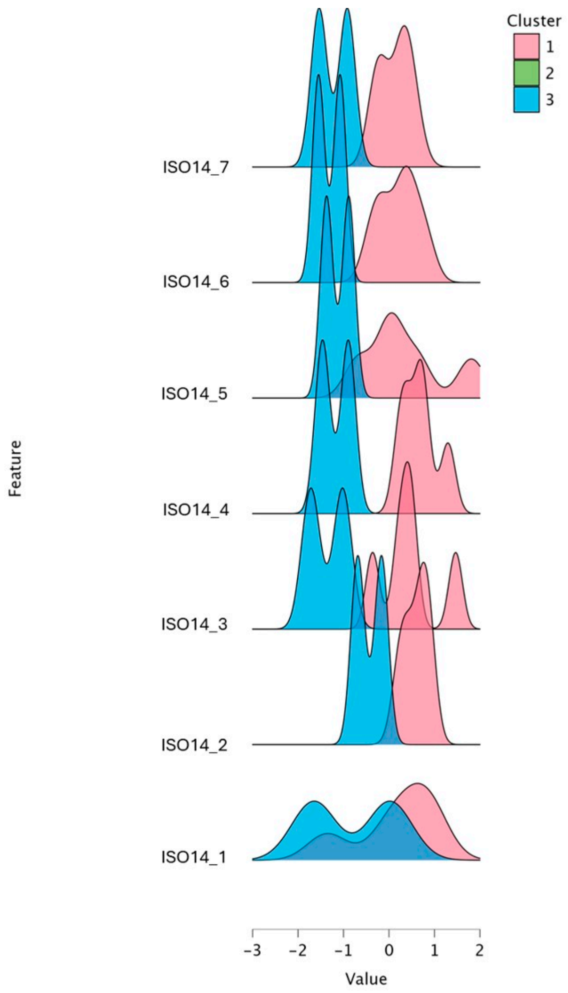

Figure S5. Distribution of the standardised biodegradation indicators used in the clustering for the ISO 14855-1:2012 assay shown separately for each cluster.

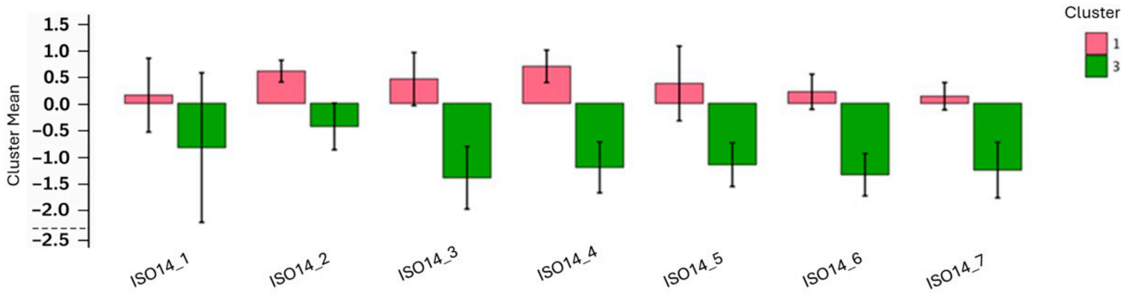

Figure S6. Cluster means of the standardised indicators for the ISO 14855-1:2012 assay.

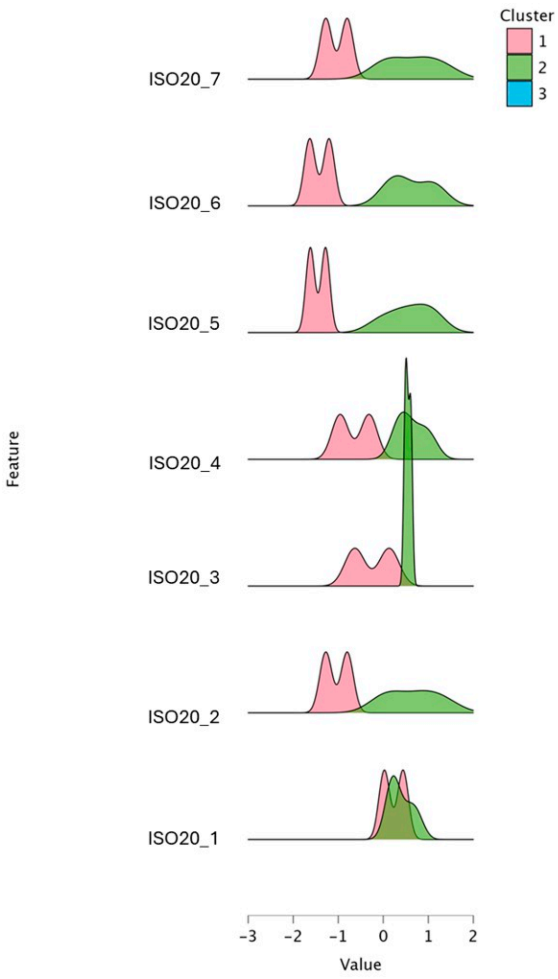

Figure S7. Distribution of the standardised biodegradation indicators used in the clustering for the ISO 20136:2020 assay shown separately for each cluster.

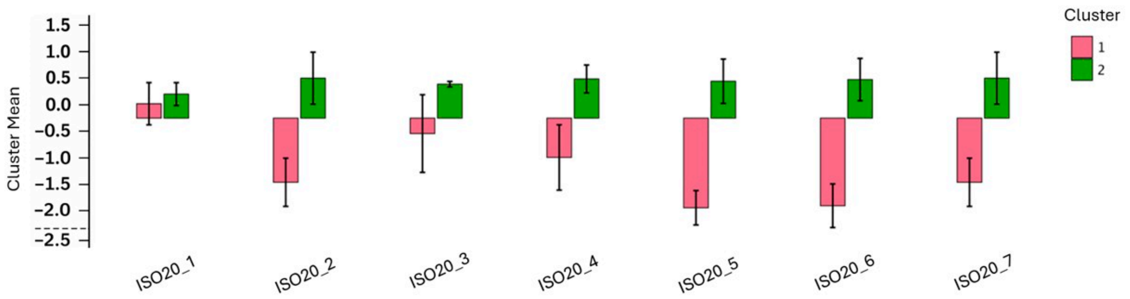

Figure S8. Cluster means of the standardised indicators for the ISO 20136:2020 assay.

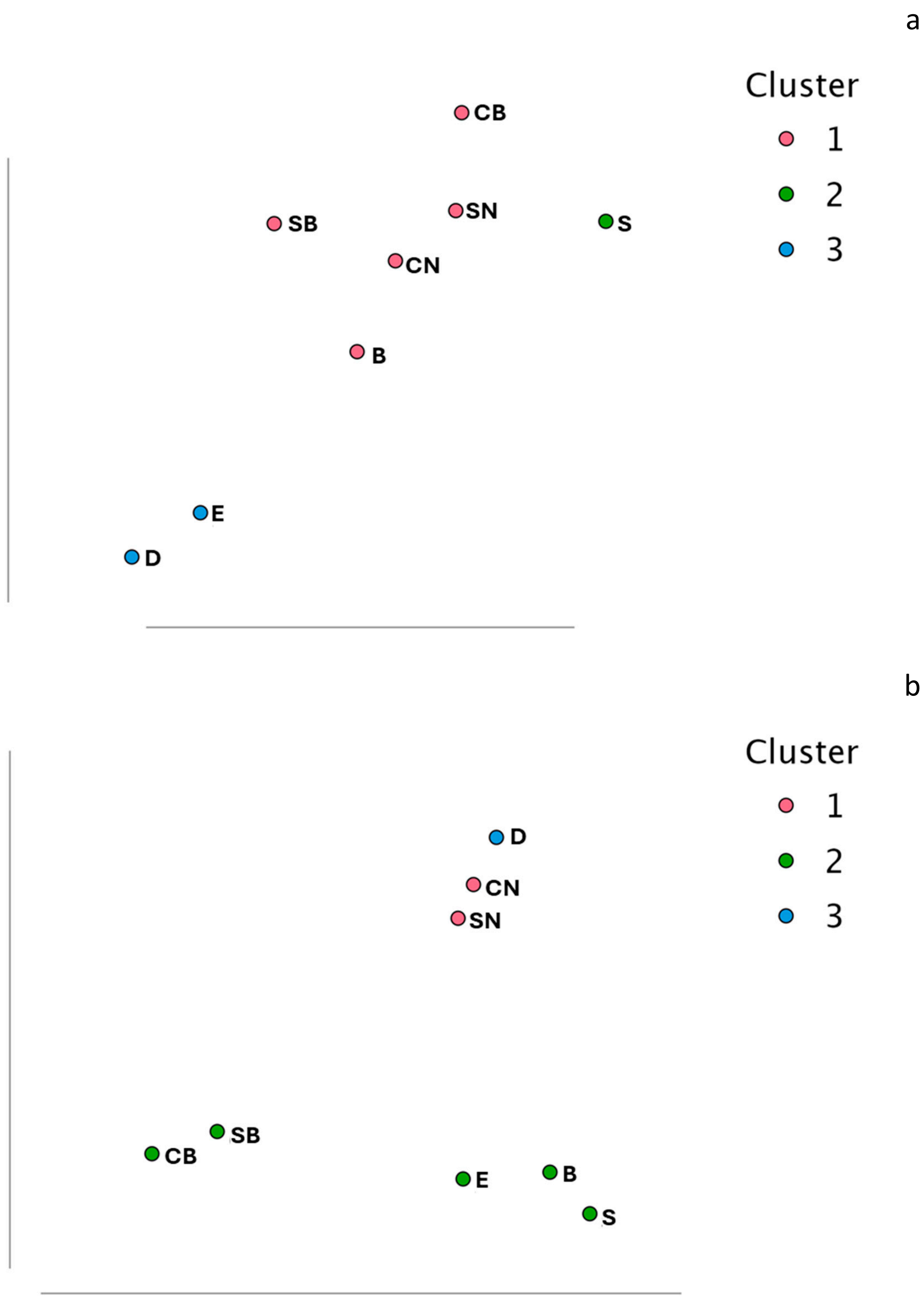

Figure S9. Complementary t-SNE visualisation of the standardised biodegradation indicators obtained for the (a) ISO 14855-1:2012 and (b) ISO 20136:2020 assays.

Table S1. Characteristics and chemical composition of the compost mature inoculum.

Sample	TC (%)	TN (%)	C/N ratio	SST (%)	SV (%)	pH	Rottegrad test	Aeration (ml/min)**	Measure frequency (min)**	Reactor mass (gr d.w)	Sample mass (gr)
Mature Compost	32.03	2.86	11.20	31.97	63.95	8.86	V	250-500	30	300	15

* Source: The compost utilized in this study was produced at an industrial scale from a feedstock mixture comprising bovine manure, olive mill wastewater, and lignocellulosic pruning residues.

** During the process.

Table S2. Characteristics and chemical composition of all the eco-friendly tanned leathers tested.

Sample	Tanning	Retanning	Finish	TC (%)	CO_2 (g)	TN (%)	C/N ratio
B	Zeolite	Retanal	Mix	43.44	23.89	8.70	4.99
E	Zeolite	Retanal	Mix	45.66	23.44	10.04	4.55
D	Aluminium	Metal free	Copolyamide	41.51	22.83	9.99	4.16
S	Titanium	Metal free	Mix	46.67	25.67	9.98	4.68
CB	Phenolic	Metal free	Mix	46.92	25.81	9.67	4.85
CN	Phenolic	Metal free	Mix	44.15	24.28	12.13	3.64
SB	Phenolic	Metal free	Mix	49.32	27.13	10.43	4.73
SN	Phenolic	Metal free	Mix	43.63	22.40	12.62	3.46

Table S3. Heavy metal, metalloid, and fluorine content in the initial leather samples and their corresponding final composts. All results are expressed in mg kg^{-1} on a dry mass basis.

Sample	Matrix	As	Cd	Cr	Cu	F	Hg	Mo	Pb	Ni	Se	Zn
B	Initial leather	<1	<0.5	36.91	<10	<LOD	<0.15	<1	<1	1.38	<0.75	89.11
B	Final compost	1.95	<0.5	19.05	71.25	<LOD	<0.2	2.30	6.20	10.20	1.40	271.20
E	Initial leather	<1	<0.5	12.47	<10	<LOD	<0.15	<1	<1	1.48	<0.75	138.2
E	Final compost	2.15	<0.5	19.45	66.70	<LOD	<0.2	2.55	6.90	11.45	1.50	276.95
D	Initial leather	<1	<0.5	8.57	<10	<LOD	<0.15	<1	<1	<1	<0.75	95.87
D	Final compost	1.80	<0.5	12.70	68.30	<LOD	<0.2	1.95	6.10	8.15	1.20	248.85
S	Initial leather	<1	<0.5	47.66	<10	<LOD	<0.15	<1	1.16	1.29	<0.75	20.36
S	Final compost	2.00	<0.5	19.50	73.80	<LOD	<0.2	2.35	6.75	10.50	1.25	275.00
CB	Initial leather	<1	<0.5	18.10	<10	<LOD	<0.15	<1	<1	<1	<0.75	81.43
CB	Final compost	0.85	<0.5	5.90	16.75	<LOD	<0.15	3.75	4.45	2.85	<0.75	40.85
CN	Initial leather	<1	<0.5	19.72	<10	<LOD	<0.15	<1	<1	<1	<0.75	<10
CN	Final compost	2.30	<0.5	16.20	76.35	<LOD	<0.15	2.80	6.25	10.65	1.35	291.50
SB	Initial leather	<1	<0.5	23.03	<10	<LOD	<0.15	<1	<1	<1	<0.75	10.56
SB	Final compost	2.20	<0.5	23.50	77.35	<LOD	<0.15	1.30	8.90	11.20	1.30	279.45
SN	Initial leather	<1	<0.5	18.12	<10	<LOD	<0.15	<1	<1	<1	<0.75	10.72
SN	Final compost	2.30	<0.5	18.20	65.35	<LOD	<0.15	1.95	5.95	9.95	1.10	232.10

Note. Measured concentrations are expressed on a dry matter basis (d.m.). Maximum reference levels established for compostability studies (mg kg^{-1}): As 5, Cd 0.5, Cr 50, Cu 50, Hg 0.5, Mo 1, Ni 25, Pb 50, Se 0.75, Zn 150 and F 100. Analytical reporting/detection limits used for the elemental analysis after acid digestion treatment (mg kg^{-1}): As 1, Cd 0.5, Cr 1, Cu 10, Hg 0.15, Mo 1, Ni 1, Pb 1, Se 0.75 and Zn 10. These reference and detection values are not measured sample concentrations. Company names, supplier identities and commercial product references are intentionally omitted in accordance with confidentiality agreements signed with the collaborating companies. Less-than signs indicate concentrations below the analytical reporting limit. For fluorine (F), <LOD indicates that all measurements were below the

instrumental limit of detection; therefore, no numerical concentration is reported. Final compost refers to the compost obtained after the ISO 14855-1:2012 composting assay. Apparent increases in some metal concentrations in the final compost should be interpreted considering both the background composition of the mature compost used as inoculum/matrix and the mass-balance nature of composting. During ISO 14855-1:2012 composting, part of the organic carbon is mineralised and released mainly as CO_2, reducing the organic mass of the system and relatively concentrating the residual inorganic fraction. Therefore, metal concentrations measured in the final compost cannot be attributed exclusively to release from the leather samples; they may reflect the combined contribution of the initial compost matrix, organic matter loss during biodegradation, relative concentration of inorganic residues and, where applicable, metals originally present in the leather.

Table S4. Interparticulate and intraparticulate porosity of pristine and treated leather samples

Sample		Porosity (%) Interparticulate	Porosity (%) Intraparticulate
B	**Pristine**	15.9	26.3
	**Aged**	6.2	3.1
D	**Pristine**	26.5	35.7
	**Aged**	18.2	12.9
E	**Pristine**	21.6	24.3
	**Aged**	5.7	7.4
S	**Pristine**	28.4	12.7
	**Aged**	20.5	7.8
CB	**Pristine**	23.3	16.5
	**Aged**	17.0	7.6
CN	**Pristine**	27.3	19.8
	**Aged**	14.2	6.9
SB	**Pristine**	18.8	17.5
	**Aged**	14.3	7.4
SN	**Pristine**	22.1	28.1
	**Aged**	14.4	5.7

Table S5. Leather composting data from ISO 14855-1:2012 for statistical analysis and features (variables) used for ISO 14855-1:2012 statistical analysis.

Sample	ISO14_1	ISO14_2	ISO14_3	ISO14_4	ISO14_5	ISO14_6	ISO14_7	Cluster	Tanning
B	76.16	76.11	76.16	76.16	27.13	55.31	76.16	1	Zeolite
E	73.03	67.19	65.54	65.34	14.60	40.96	67.22	3	Zeolite
D	90.92	59.01	71.00	59.92	7.95	34.06	59.13	3	Aluminium
S	100.04	36.64	82.94	63.90	32.51	79.42	102.69	2	Titanium
CB	100.01	80.13	90.45	86.28	51.39	67.92	86.04	1	Phenolic
CN	90.44	83.11	80.60	80.62	27.12	62.32	83.20	1	Phenolic
SB	100.00	73.30	82.13	77.82	17.38	51.52	76.27	1	Phenolic
SN	94.89	82.72	83.01	81.03	35.37	61.62	82.80	1	Phenolic

ISO14_1	Final biodegradation value
ISO14_2	Biodegradation at 1/3 of the total test time
ISO14_3	Biodegradation at 2/3 of the total test time
ISO14_4	Biodegradation at 1/2 of the total test time
ISO14_5	Time required for each sample to reach 25% of its maximum biodegradation
ISO14_6	Time required for each sample to reach 50% of its maximum biodegradation
ISO14_7	Time required for each sample to reach 75% of its maximum biodegradation

Table S6. Leather liquid-phase biodegradation data from ISO 20136:2020 for statistical analysis and features (variables) used for ISO 20136:2020 statistical analysis.

Sample	ISO20_1	ISO20_2	ISO20_3	ISO20_4	ISO20_5	ISO20_6	ISO20_7	Cluster	Tanning
B	86.86	63.61	86.52	81.26	7.83	12.19	63.61	2	Zeolite
E	87.80	58.06	83.80	75.56	7.13	10.83	58.06	2	Zeolite
D	25.27	24.67	25.27	25.27	6.02	9.36	24.67	3	Aluminium
S	86.34	70.20	86.34	85.68	6.02	11.36	70.20	2	Titanium
CB	99.69	45.93	84.86	70.65	9.15	14.60	45.93	2	Phenolic
CN	82.58	22.54	59.86	44.52	2.90	6.06	22.54	1	Phenolic
SB	94.98	47.89	84.01	72.31	8.80	14.12	47.89	2	Phenolic
SN	92.35	31.08	76.18	57.67	2.02	4.52	31.08	1	Phenolic

ISO20_1	Final biodegradation value
ISO20_2	Biodegradation at 1/3 of the total test time
ISO20_3	Biodegradation at 2/3 of the total test time
ISO20_4	Biodegradation at 1/2 of the total test time
ISO20_5	Time required for each sample to reach 25% of its maximum biodegradation
ISO20_6	Time required for each sample to reach 50% of its maximum biodegradation
ISO20_7	Time required for each sample to reach 75% of its maximum biodegradation
